# Assessing the inflammatory response to in vitro polymicrobial wound biofilms in a skin epidermis model

**DOI:** 10.1038/s41522-022-00286-z

**Published:** 2022-04-07

**Authors:** Jason L. Brown, Eleanor Townsend, Robert D. Short, Craig Williams, Chris Woodall, Christopher J. Nile, Gordon Ramage

**Affiliations:** 1 Oral Sciences Research Group, Glasgow Dental School, School of Medicine, College of Medical, Veterinary and Life Sciences, University of Glasgow, Glasgow, G12 8TA UK; 2 Glasgow Biofilm Research Network, 378 Sauchiehall Street, Glasgow, G2 3JZ UK; 3 School of Life Sciences, Gibbet Hill Campus, The University of Warwick, Coventry, CV4 7AL UK; 4 Department of Chemistry and Material Science Institute, University of Lancaster, Lancaster, LA1 4YB UK; 5 Microbiology Department, Lancaster Royal Infirmary, University of Lancaster, Lancaster, LA1 4YW UK; 6 Blutest Laboratories, 5 Robroyston Oval, Nova Business Park, Glasgow, G33 1AP UK; 7 School of Dental Sciences, Newcastle University, Newcastle, NE2 4BW UK

**Keywords:** Biofilms, Antimicrobials

## Abstract

Wounds can commonly become infected with polymicrobial biofilms containing bacterial and fungal microorganisms. Microbial colonization of the wound can interfere with sufficient healing and repair, leading to high rates of chronicity in certain individuals, which can have a huge socioeconomic burden worldwide. One route for alleviating biofilm formation in chronic wounds is sufficient treatment of the infected area with topical wound washes and ointments. Thus, the primary aim here was to create a complex in vitro biofilm model containing a range of microorganisms commonly isolated from the infected wound milieu. These polymicrobial biofilms were treated with three conventional anti-biofilm wound washes, chlorhexidine (CHX), povidone-iodine (PVP-I), and hydrogen peroxide (H_2_O_2_), and efficacy against the microorganisms assessed using live/dead qPCR. All treatments reduced the viability of the biofilms, although H_2_O_2_ was found to be the most effective treatment modality. These biofilms were then co-cultured with 3D skin epidermis to assess the inflammatory profile within the tissue. A detailed transcriptional and proteomic profile of the epidermis was gathered following biofilm stimulation. At the transcriptional level, all treatments reduced the expression of inflammatory markers back to baseline (untreated tissue controls). Olink technology revealed a unique proteomic response in the tissue following stimulation with untreated and CHX-treated biofilms. This highlights treatment choice for clinicians could be dictated by how the tissue responds to such biofilm treatment, and not merely how effective the treatment is in killing the biofilm.

## Introduction

Chronic wounds such as diabetic foot ulcers (DFU) provide a substantial socioeconomic burden globally. It is predicted that almost 1% of the NHS budget per year is spent on health care costs towards ulceration and amputation in DFU, with an estimate of between £837 and £962 million being spent annually to combat the disease^1^, whilst on wounds in general it has been reported that the cost could well exceed £4.5 billion^2^. Further to the financial burden associated with the disease, patients with chronic non-healing wounds have high morbidity and mortality rates. An alarming statistic from a recent report documented that the mortality rate for people who undergo a major lower extremity amputation caused by DFU will be dead within 5 years^3^. Although a range of environmental and host-related genetic factors play a role in the pathophysiology of DFUs, infections of the wound sites are one of the main reasons why morbidity and mortality rates are so high. Infection of DFUs in patients can range from 40 – 60%^4–7^, and it is the capacity of different microorganisms to colonize the wound bed that can prevent sufficient clinical management of the DFUs and other chronic wounds^8^.

Clinically, wounds can present as acute or chronic depending on their nature of healing; acute wounds tend to process through the normal rate of healing (days to weeks), whilst chronic wounds are generally defined as those that are unable to heal appropriately after a period of between 4 weeks to 3 months^9^. As discussed briefly above, a major reason whereby such chronic wounds are unable to heal is partly due to the accumulation of microorganisms in the wound bed, forming biofilm aggregates on the skin surface deep within the ulcerated tissue. Indeed, a meta-analysis of published data indicated a 78.2% prevalence of biofilms present in chronic wounds^10^. Interestingly, James et al (2008) showed similar results using specimens from subjects with chronic and acute wounds, with 60% of the chronic wound samples containing polymicrobial communities of aerobic and anaerobic bacteria, but only one of the 16 acute wound samples containing a biofilm^11^. Nowadays due to advancements in molecular technologies, we are starting to appreciate the fundamental role that fungi might play in the microbial landscape of the wound^12–14^. It is clear from these studies that the role of fungi and bacteria needs to be investigated further to truly understand how these organisms interact together and interfere with the process of wound healing.

Existing “models” for wound biofilms are often limited to a few microorganisms and fail to incorporate other important characteristics within the model system including a suitable substratum, culture media, and incubation conditions, amongst others^15,16^. At this juncture, our research group has published a study looking at a simplistic triadic interkingdom biofilm model containing *Pseudomonas aeruginosa* and *Staphylococcus aureus*, two co-colonizers of chronic wounds^17^, and *Candida albicans* grown in microtiter plates (e.g., 2D) and on a 3D hydrogel substratum. It was found that the more representative 3D biofilm substrata exhibited greater resistant to antimicrobial wound washes compared to those biofilms grown on 2D plastic surfaces, suggestive that the model system used is fundamental to truly mimic the in vivo environment^18^. The same model was utilised to assess a combination of antibacterial and antifungal therapies to combat the mixed-species biofilm consortia, highlighting the importance of a fungal element in increasing antimicrobial resistance in the biofilm community^19^. However, as with most existing models this consortium was limited to three microorganisms, without including anaerobic species that are often isolated from the hypoxic or anoxic niche of chronic wounds and particularly DFUs^20^. Therefore, there remains a need for creating a more complex consortium model containing a variety of wound-associated microorganisms including both bacterial and fungal entities.

In certain patients, an infected chronic wound will fail to repair and heal even weeks to months following microbial colonization. This has been shown extensively in vivo using either porcine, murine or rabbit models of wound healing. These model systems tend to involve incorporating *S. aureus* and/or *P. aeruginosa* into an artificial wound, often induced through a burn, biopsy punch or similar. Generally, biofilm formation within these wound lead to significant delays in wound healing and closure^16,21^. Similar in vivo models have also been used effectively to evaluate the role of polymicrobial biofilms in wound healing showing similar results to mono-species studies^22,23^. Interestingly, the work Dalton et al (2011) showed that wound closure in a chronic wound murine model was slightly more delayed following infection with a 5-species polymicrobial consortia than with *P. aeruginosa* planktonic cells only^22^. At this juncture, a recent study showed that the transcriptional profile of DFU tissues are unique to disease severity^24^, suggestive that microbial bioburden in the wound can impact the host response. Ex vivo and in vitro models have also been reported in the literature as effective systems for studying host-pathogen interactions^16,25^. Of those in vitro models that do exist most are again limited in the microbial consortia used, with many restricted to assessing the host response in tissue to singular microorganisms^26–30^.

The purpose of the study is three-fold; firstly, to optimize the creation of a polymicrobial biofilm containing aerobes, anaerobes, and fungi associated with wound microbiomes. Secondly, the model will be used for testing three conventional topical wound washes (chlorhexidine, povidone iodine and hydrogen peroxide) in a 3D-hydrogel-based cellulose substrate system. Finally, the treated and untreated biofilms will be utilised for assessing the inflammatory response in a 3D skin epidermis, results of which may dictate which therapeutic could serve as the best candidate for antibiofilm activity in wound treatment. This model attempts to recapitulate the complex interkingdom nature of the wound microenvironment in an 3D in vitro setting.

## Results

**Fig. 1:**
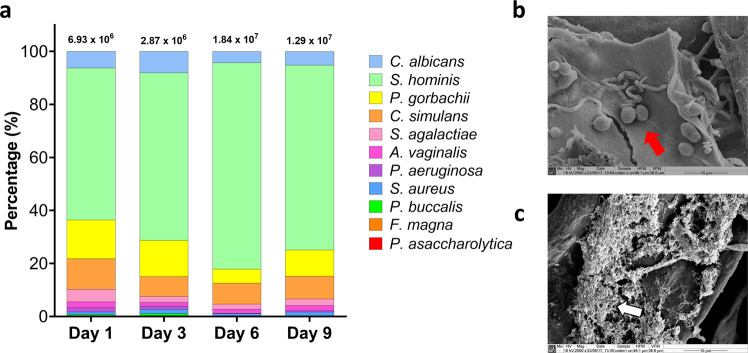
Compositional and microscopic analysis of the 11-species biofilms grown under anaerobic conditions. A total of 10 bacterial species and 1 fungal species were cultured under anaerobic conditions for a total of 9 days. On days 1, 3, 6, and 9, biofilms were sonicated, and DNA extracted. Compositional analysis was achieved using species or genus-specific primer sets and qPCR (**a**). Biofilms at day 1 and day 9 were imaged using scanning electron microscopy at a magnification of x2500. Fungal yeast cells are highlighted by the red arrow in panel **b**, whilst the thick extracellular matrix in the mature biofilm is indicated using white arrows in panel **c**. Data for compositional analysis is presented as mean % composition on a log scale for visualisation of all microorganisms. These mean values are taken a total of 2 independent experiments, containing 3 replicates. A more detailed depiction of the % compositional changes in the less abundant microorganisms can be seen in Supplementary Fig 2. CFE/mL values with standard deviation for each microorganism for each condition and across all timepoints are shown in Supplementary Fig 3.

Firstly, four timepoints and three incubation conditions were selected to assess the development of early and mature wound biofilms on the cellulose matrix as a substratum. The fibrillar nature of the matrix can be seen in Supplementary Fig 1. Multiple timepoints and different incubation environments were utilized to evaluate compositional changes over 9 days, and for the purposes of selecting the optimal culture time and conditions. Compositional qPCR analyses were used to assess the proportion of the 11 microorganisms in the complex wound model. Using species/genus-specific primers, each microorganism was identified in the biofilm from 24 h to 9 days of growth under anaerobic, aerobic and 5% CO_2_ conditions. As anticipated, there was clear variation in the level of colonization of each microorganism in the biofilms grown in different atmospheric conditions. In particular, at all timepoints microbial diversity increased in the biofilms grown in AnO_2_ conditions, with four of the five anaerobic microorganisms (*P. asaccharolytica*, *F. magna*, *P. buccalis*, and *A. vaginalis*) generally increasing in proportion when grown in the absence of oxygen (Fig. 1a). In the biofilms grown in 5% CO_2_ and in aerated conditions, *C. albicans* predominated the microbial consortia, comprising between ~30% and ~75% of the total bioburden (Supplementary Fig 2). However, under anaerobic environments, the proportion of the fungal element in the total composition ranged from between ~4% and ~10% (Fig. 1a), which is in line with the skin mycobiome, which consists of <10% of the total microbial population at the skin epithelial surface^31^. All culture conditions and timepoints gave reproducible results across experiments (Supplementary Fig 3). Given the results highlighted above and the hypoxic nature of the wound bed^32–34^, AnO_2_ conditions were selected for biofilm growth moving forward with this study.

For the AnO_2_ conditions specifically, CFE/mL for these organisms increased from 6.93 ×10^6^ CFE/mL microorganisms in the 24 h-mature biofilm, to 1.29 ×10^7^ CFE/mL in the 9-day biofilm (Fig. 1a). For all timepoints, *S. hominis* remained the most abundant organism, ranging from ~55% to ~75% of the total microbial consortia. SEM imaging highlighted the porous nature of the CM material (Supplementary Fig 1), whilst in the microbial-inoculated CM, there is a sparse covering of the matrix surface with the biofilm at the earlier timepoint, with bacteria largely indiscernible on the substrate (24 h; Fig. 1b). In addition, it is clear from the SEM imaging that *C. albicans* yeast was attached to the CM, highlighted by the white arrows (Fig. 1b) with subtle evidence of hyphal formation. At the later timepoint (9 days), a dense biofilm is visible with thick extracellular matrix (Fig. 1c). From this data, it can be concluded that we have successfully developed an early and mature wound biofilm model, with sufficient colonization of aerobic and anaerobic bacteria, including a common skin fungal organism.

**Fig. 2:**
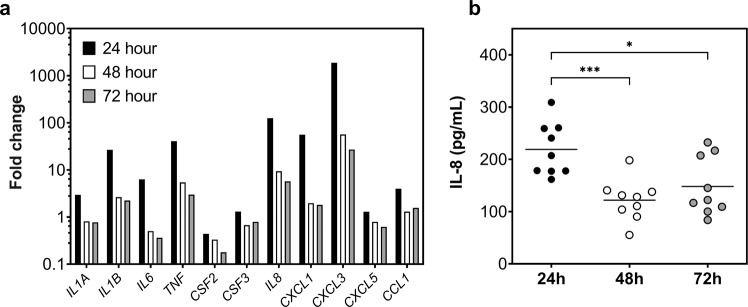
Inflammatory response in differentiated THP-1 monocytes following exposure to early and more mature biofilms. THP-1 monocytes (1 ×10^6^ cells/mL) were differentiated using vitamin D3 then exposed to conditioned media from 24-, 48- and 72-h biofilms for 24 h at 37 °C, 5% CO_2_. Following incubation, RNA was extracted from the THP-1 cells, converted into cDNA then used in a qPCR profiler array to assess gene expression. Data presented as fold change relative to unstimulated controls (**a**). Spent media from the co-culture was also assessed for IL-8 levels using a sandwich ELISA method (**b**). Statistical significance was determined using one-way ANOVA with Tukey’s post-test (*p < 0.05, *** *p* < 0.001).

Next, for the purposes of preliminary assessment of the host response, gene expression of inflammatory markers and IL-8 production was assessed in THP-1 monocytes following exposure to early and late biofilm model spent media. For this, differentiated monocytes were co-cultured with conditioned spent media from biofilms grown for 24-, 48-, and 72- h. The transcriptomic response was assessed using a custom RT_2_ profiler array, and the IL-8 protein concentration were determined using ELISA. The results from this part of the study indicated that the 24-h biofilm supernatant elicited the greatest change in expression of pro-inflammatory mediators, as well as increasing the production of IL-8 by the monocytes (Fig. 2). Specifically, expression of proinflammatory cytokines, *IL1β*, *TNF*, and *IL8* were all increased by 26-fold, 40-fold, and 125- fold, respectively, compared to the untreated controls. Interestingly, expression of all genes assessed was higher in monocytes stimulated with 24 h biofilm spent media, than those exposed to 48-h and 72-h biofilm supernatants (Fig. 2a). At the protein level, the concentration of IL-8 released by the cells was significantly higher in those stimulated with 24-h biofilm media, compared to 48-hour (***p < 0.001), and 72 h (*p < 0.05), respectively (Fig. 2b). It is noteworthy that no significant differences in THP-1 cell viability was detected across all timepoints (data not shown). Taken together, these results indicate that the 24 h biofilm is more proinflammatory than the more mature biofilms (48-h and 72-h biofilms). Thus, the 24-hour biofilm model was selected for the following 3D co-culture model system with skin epidermis tissue.

**Fig. 3:**
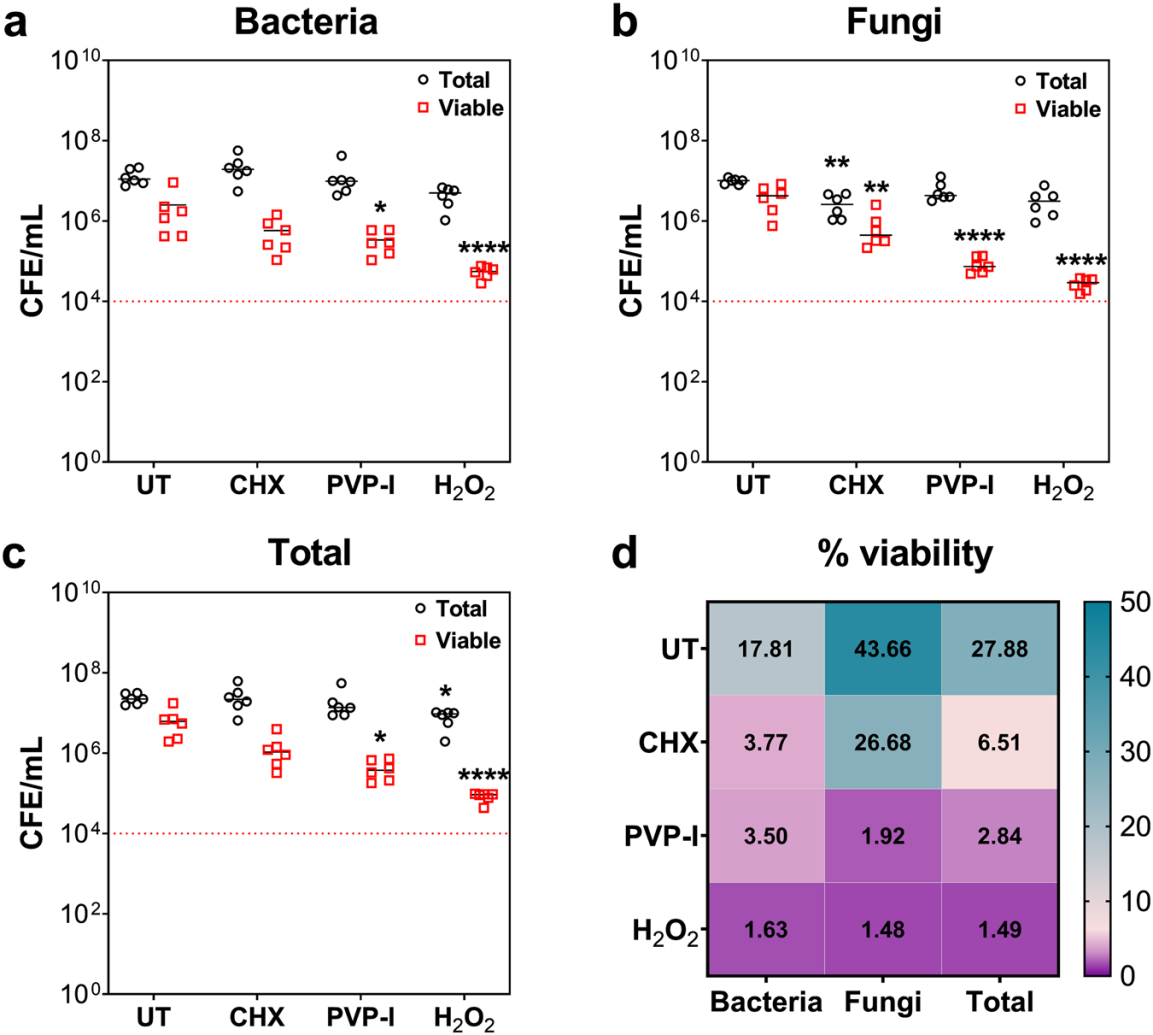
Testing the efficacy of the three anti-biofilm wound washes. 24-hour biofilms were treated with 3% H_2_O_2_, 10% w/v PVP-I, and 0.05% CHX for 24 hours at 37^o^C under anaerobic conditions. Following treatment, viability of the biofilms were assessed using live/dead qPCR analyses for total bacterial counts (16 S; **a**), total fungal counts (18 S; **b**) and combined counts (**c**). % viability was calculated using the above counts for total vs. viable cells (**d**). Statistical significance was determined using a two-tailed Student’s t-test to compare the means of treated biofilms vs untreated biofilms (* *p* < 0.05, ** *p* < 0.01, **** *p* < 0.0001).

The purpose of the following part of the study was to assess the skin epidermis response to untreated and antiseptic-treated biofilms. Firstly, the efficacy of three anti-biofilm wound washes (CHX, PVP-I, and H_2_O_2_) was tested against the 24-hour biofilm (Fig. 3). Of these three treatment modalities, H_2_O_2_ was the most effective in reducing the viability of the bacteria (Fig. 3a), fungi (Fig. 3b), and total microbes (Fig. 3c; all ****p < 0.0001) when compared to untreated controls. It should be noted that H_2_O_2_ was the only treatment that significantly reduced the total bacterial bioburden. Bacterial numbers were reduced from ~1.33 ×10^7^ CFE/mL to ~4.45 ×10^6^ CFE/mL when biofilms were treated with H_2_O_2_ (Fig. 3a), whilst fungal numbers were decreased from 9.98 ×10^6^ CFE/mL to 3.39 ×10^6^ CFE/mL (Fig. 3b). Although CHX significantly reduced the total fungal load to 2.78 ×10^6^ CFE/mL, it was the least effective of the three treatments when relation to % viability. The treatment lowered the viability of the biofilm from 17.81% to 3.77% for bacteria, 43.66% to 26.68% for fungi, and 27.88% to 6.51% for total microbes (Fig. 3d).

**Fig. 4:**
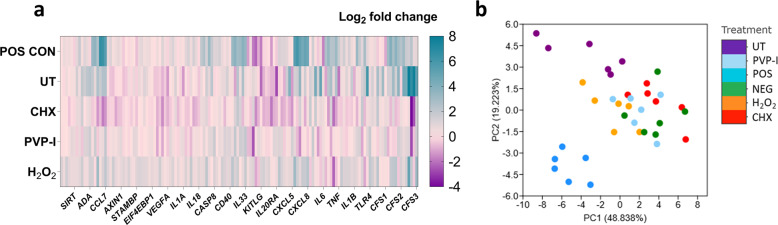
Transcriptional profiling of the RHE tissue following biofilm stimulation. Skin epidermis was exposed to treated and untreated biofilms for 24 h at 37°C, 5% CO_2_. Appropriate negative and positive controls were used. Following stimulation, RNA was extracted, cDNA synthesized, and gene expression was profiled using a custom array containing primers for different pro-inflammatory genes and upregulated proteins from the Olink technology (Fig. 5). Panel **a** depicts the Log_2_ fold change in RHE tissue stimulated with PMA (POS CON), untreated biofilms (UT), CHX-treated, PVP-I-treated, or H_2_O_2_-treated biofilms, all relative to unstimulated negative controls. Data presented as a *n* = 6, encompassing three technical replicates from two independent experiments. Each column represents these 6 replicates. Panel **b** shows a principal component analysis plot showing distinct clustering of the transcriptional profiles in the tissue following stimulation.

Next, the untreated and treated biofilms were incorporated into the 3D skin epidermis model. Biofilms were grown and treated as above, then co-cultured with reconstructed human epidermis (RHE) for 24 hours. Histological assessment of the RHE tissue revealed a multi-layered epithelial structure with keratinised peripheral cell layer, similar to that of skin epidermis in vivo (Supplementary Fig 4). Once the histological accuracy of the tissue was confirmed, the proteomic response was next assessed, results of which directed the transcriptional assessment using an RT_2_ profiler array, which contained a range of inflammation-associated genes (Fig. 4). Of these genes assessed, as expected, the greatest response was seen in the PMA-stimulated samples (positive control). This was evident from the heatmap (Fig. 4a) and the multivariate principal component analysis (PCA) plot (Fig. 4b) whereby positive controls samples showed the greatest variance from the other samples at both PC1 (~48%) and PC2 (~19%). whereby positive controls samples showed the greatest variance from the other samples at both PC1 (~48%) and PC2 (~19%). Tissue stimulated with untreated biofilms also showed some variance from other samples, clustering away from RHE stimulated with the three treated biofilms and the negative controls. It is noteworthy that this variation was also observable when positive controls were removed from the analyses, as can be seen in Supplementary Fig 5. No variance was seen in the different treatment modalities and the unstimulated control. Expression profiles for all genes are documented in the Supplementary Fig 6. Taken together, at a transcriptional level, all treatment modalities reduced the inflammatory response in the tissue to levels comparable with unstimulated RHE.

**Fig. 5:**
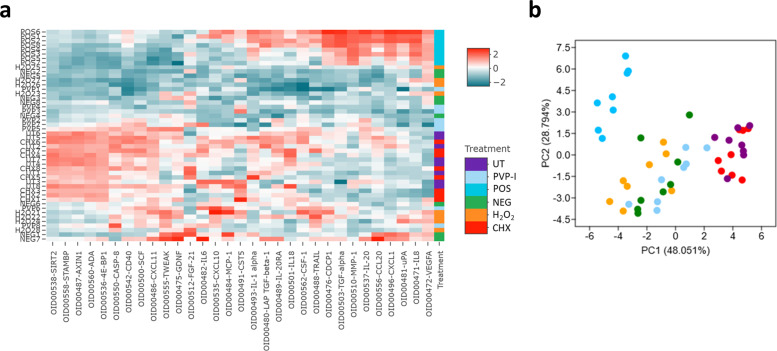
Biofilm treatment dictates the proteomic profile of the RHE tissue following co-culture. Hierarchical clustering of the proteomic response in the skin epidermis is depicted in panel **a**. For this analysis, all assay distributions in the data were centred around using NPX values for each protein, as detected using Olink technology. In brief, red bars indicate increased levels of proteins, with blue bars showing decreased levels of a given protein. A principal component analysis plot is shown in panel **b**, highlighting the unique clustering of the PMA-stimulated samples across both PC1 and PC2, with distinguishable clusters of the untreated and CHX-treated biofilms-stimulated tissue samples on PC1.

Using Olink technology and an “inflammation” panel of proteins, hierarchical clustering of the RHE protein responses to untreated and treated biofilms highlighted key differences between the treatments and controls. Firstly, in all positive controls, PMA-stimulated tissue gave rise to a unique protein response, with elevated levels of CDCP1, TGF-alpha, MMP-1, IL-20, and CXCL1 (Fig. 5a) as well as classical pro-inflammatory markers such as IL-8 and VEGFA, whereby higher protein levels were further confirmed using ELISA (Supplementary Fig 7a and b). Similarly to the transcriptional response, PCA plots highlighted that PMA-stimulated RHE samples showed the greatest variance from the other samples at both PC1 (~48%) and PC2 (~28%) (Fig. 5b). For the biofilm-stimulated tissue, the protein responses in the RHE exposed to untreated biofilms and biofilms treated with CHX clustered together, with similar protein levels for SIRT2, STAMBP, AXIN1, ADA, and 4E-BP1 (Fig. 5b). Comparable levels of the pro-inflammatory protein, IL-18 were also seen in untreated biofilm- and CHX-treated biofilm-stimulated tissue (Supplementary Fig 7c). Similar clustering of untreated biofilm and CHX-treated biofilm samples were seen using the PCA plot, with the greatest variance from other samples seen on PC1 (Fig. 5b). There were no obvious similarities seen in hierarchical clustering of the protein responses in the unstimulated control or RHE stimulated with H_2_O_2_- or PVP-I-treated biofilms, which was likely due to a higher variation in the replicates (Fig. 5a). This was further confirmed by the PCA plot, with a greater spread for each individual sample across PC1 and PC2. Nevertheless, these three variables were all relatively comparable, and clearly clustered away from the other tested parameters (Fig. 5b). Similarly to the gene expression results, these observations were also evident in the PCA plot when the positive controls were removed from the analysis, with clear clustering of the untreated biofilms and CHX-treated biofilm RHE tissue samples (Supplementary Fig 8). Overall, this highlights that H_2_O_2_ or PVP-I was the most effective treatment in reducing the otherwise elevated protein response back to levels analogous with the negative control, unstimulated tissue. Conversely, CHX-treated biofilms and untreated biofilms gave rise to unique “pro-inflammatory” protein profiles in the RHE tissue.

**Fig. 6:**
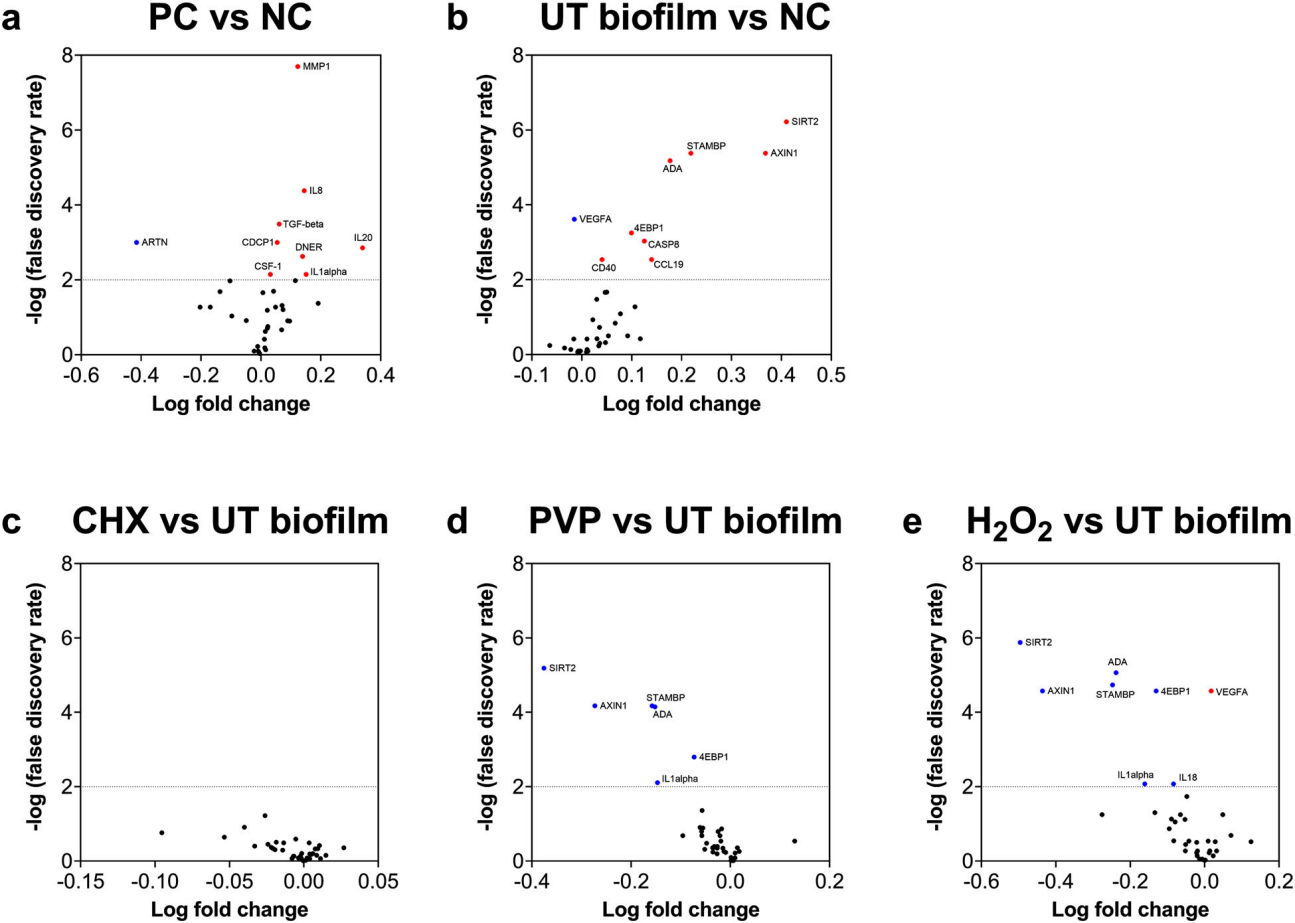
Volcano plot showing significantly down- or up- regulated proteins in RHE tissue when comparing treatment modalities with controls. Volcano plots show the log fold change in protein concentration after correction of the p values using Benjamini–Hochberg false discovery rate (FDR) of 5%. Proteins that are significantly downregulated are shown in blue, and those upregulated are red. The dotted line depicts significance value of *p* < 0.01 (-log 2.0). PMA-stimulated positive controls (PC) were first compared with unstimulated control tissue (NC) (**a**), then tissue stimulated with untreated biofilms with negative controls (**b**). Each treatment modality was then compared with untreated biofilms (CHX vs UT, panel **c**; PVP-I vs UT, panel **d** and H_2_O_2_ vs UT, panel **e**).

To further evaluate the unique protein responses in the tissue, statistically significant changes in the levels of proteins between tissue exposed to either treated or untreated biofilms were assessed using a volcano scatter plot (Fig. 6). For our internal positive controls, a total of 8 proteins were significantly elevated in RHE stimulated by PMA when compared to unstimulated tissue, whilst one was decreased in concentration (Fig. 6a). Of these proteins, the levels of IL-20 were the greatest change. When comparing the tissue stimulated by an untreated biofilm with the negative controls, a total of 8 proteins were increased, with one significantly lower (Fig. 6b). Of the proteins increased in concentration, the greatest changes were observed for SIRT2, AXIN1, and STAMBP, which are all associated with intracellular inflammatory pathways and cell cycle biological systems. VEGFA levels were significantly reduced in biofilm-stimulated tissue, which corroborates with the observations shown in Supplementary Fig 7b. Finally, to assess whether any significant changes were observable between tissue stimulated with treated or untreated biofilms, statistically significant proteins were plotted against fold change for CHX-, PVP-I-, or H_2_O_2_- treated biofilms vs. untreated biofilms. As expected from earlier results, no proteins were significantly altered in concentration from tissue subject to stimulation by untreated biofilms or CHX-treated biofilms (Fig. 6c), suggestive that the proteomic response in the tissue is comparable following exposure to untreated or CHX-treated biofilms. However, PVP-I-treated biofilms significantly reduced the levels of 6 proteins from the RHE tissue when compared to untreated biofilms (Fig. 6d), whilst H_2_O_2_-treated biofilms decreased a total of 7 proteins, causing an increase in one protein, VEGFA, when compared to negative control biofilms (Fig. 6e). Taken together, this shows that the treatment modality of biofilms can influence the level of inflammation in the RHE tissue in vitro, particularly at the protein level.

## Discussion

This study has highlighted a unique series of experiments using different 3D co-culture model systems to assess the efficacy of anti-wound washes for the treatment of polymicrobial wound biofilms. Following the creation of a complex polymicrobial biofilm model containing a range of commonly identified aerobes, anaerobes, and a fungal species on the skin surface and in the infected wound bed, we demonstrated that all three anti-wound washes, CHX, PVP-I, and H_2_O_2_, were suitable in reducing the viability of the biofilm. The treatment modality then affected the RHE tissue transcriptional and proteomic response to the biofilms, with CHX-treated and untreated biofilms driving unique protein responses in the skin epidermis. Conversely, PVP-I and H_2_O_2_ treatment were effective in alleviating the inflammatory response in the tissue at both the gene and protein level. Therefore, the results from this study show that treatment modality may influence the effective treatment of chronic wounds, including appropriate tissue response and repair following perturbation of the skin barrier by the microorganisms.

In vitro biofilm models that truly recapitulate the complex nature of the polymicrobial environment of the wound bed are limited. Existing models are simplistic in nature and often fail to incorporate multiple microorganisms and other important characteristics such as culture media including appropriate host nutrients^15,16^. Those models that do incorporate multiple microbial species, tend to overlook the importance of including a pathogenic fungal element; *Candida* spp. are commonly identified in wounds, whilst several other fungal species have also been reported to inhabit the wound bed^13,14,35,36^. Therefore, we wanted to ensure that the wound biofilm model used in this study would include a fungal pathogen, *C. albicans*. It was also pertinent to include such an organism given its ability to structural stabilise the biofilm community through its size and enhance antimicrobial resistance within the community^18,19,37–39^. However, as with any biofilm model system there remains caveats. Indeed, it has recently been shown that *P. aeruginosa* transcriptomes differ considerably in expectorated cystic fibrosis sputum vs. animal or in vitro grown models^40^, suggestive that although the organisms are present their function may differ when grown in the laboratory. Furthermore, experimental factors such as substrata and media used can influence biofilm attachment, growth, and composition^25^. Here, the biofilms were formed on cellulose matrix as the substrata, to mimic wound dressing, with the serum-rich hydrogel platform providing the nutrient source. Future studies may merit the assessment of other substrates and media as potential alternatives. In particular, direct growth of the biofilm on the RHE should be considered, to more closely recapitulate the formation of a biofilm on skin tissue. Nevertheless, we believe such a biofilm as described here far exceeds similar models in polymicrobial accuracy and complexity.

We initially selected a range of incubation conditions to culture the complex biofilm models whilst assessing their compositional changes. It is well documented that the infected wound bed is hypoxic or anoxic in nature^32–34^, providing an excellent microenvironment for anaerobic organisms to flourish. However, depending on the severity and depth of the wound, oxygen saturation levels can fluctuate across the wound bed^41^, which could impact the level of oxygen availability to the organisms colonized on the tissue. Furthermore, it has been shown that the biofilm depth of different bacterial isolates from human chronic wounds can impact the oxygen concentration profile, whereby the thicker the biofilm the lower the pO_2_ levels, and vice versa^33^. More so, the microbial composition of the biofilm can be influenced by the consortia included, with anaerobic organisms possessing the ability to survive under oxic conditions when grown with certain aerobic bacterial and fungal species^22,42^. Thus, the polymicrobial biofilms in this study were cultured under different conditions to initially assess the ability of the consortia to survive and establish themselves within the population. Indeed, due to the experimental set-up, it is unknown whether some of the microorganisms (e.g., the anaerobes) merely attached to the substrata and persisted, or whether they grew whilst in the polymicrobial consortia. Investigation of this goes far beyond the scope of this study, but would be of interest, nonetheless.

Here, under aerobic or 5% CO_2_ conditions, *C. albicans* predominated the biofilm at levels far above those expected in the mycobiome of the wound (>30%). Using the qPCR methodology, we were unable to determine whether *C. albicans* was present in its hyphae or yeast morphology, a phenotype, which would impact the organisms ability to persist and support the bacterial community. Nevertheless, it is apparent from the SEM imaging that *Candida* hyphal formation was present even after 24 hours suggestive of a structural network for bacteria as the biofilm maturity increases. In vivo, a publication by Oh et al., (2014) highlighted elegantly the biogeography of the human skin, and that such a niche consisted of mostly bacteria with fungi comprising a relatively small proportion of the total microbiota (<10%), a phenomenon that varied depending on body site^31^. Interestingly, it was *Malassezia* spp. which were found to be the most prevalent fungal species at the skin surface, which has been further confirmed elsewhere^43^, although this is again, dependent on the body site niche. *Malassezia* spp. are lipophilic in origin they require a rich lipid source e.g., sebum produced by sebaceous glands, which are located throughout the body except for the hands and soles of the feet. Thus, the mycobiome of different foot sites is rather unique e.g., plantar heel, toenail, and toe web as they have a much higher fungal diversity than elsewhere, including different *Candida* spp.^31,43^. Thus, although this model may be limited in it’s mycobial complexity, it provides a useful interkingdom testing platform for future antimicrobial studies.

Prior to assessing the inflammatory response in the RHE tissue to the treated and untreated biofilms, the efficacy of the three-candidate anti-wound washes were tested within the 3D hydrogel system. Antiseptic wound washes are commonly used to treat chronic wound infections such as DFU, either alone or to augment antimicrobial therapy, with iodine solutions, chlorhexidine, and hydrogen peroxide being common choices alongside alcohol washes and triclosan^44–46^. Publications directly comparing the effectiveness of these three washes are limited, and those that do exist are restricted to mono-species biofilm research. For example, PVP-I was the most inferior of the three wound washes (3% H_2_O_2_, 10% PVP-I, and a commercial mixture of alcohols/CHX) in reducing the biomass of a *Staphylococcus epidermidis* biofilm, even after 60 minutes of treatment, although the viability of the biofilm was reduced^47^. Others have shown their efficacy against *S. aureus*, *P. aeruginosa* or *S. epidermidis* mono-species biofilms both in vitro and in vivo^48–50^. A study investigating a multi-species wound consortium of six microorganisms revealed iodine-based dressings completely disrupted established 7-day biofilm, although only levels of *S. aureus* and *P. aeruginosa* were quantifiable within the model which is an obvious limitation^51^. Nevertheless, it is clear from the above studies that, to the authors knowledge, none exist directly comparing the efficacy of the three antiwound washes used in this study against multi-species polymicrobial biofilms. From our results it appears that all three treatment regimens were suitable candidates in reducing the viable bioburden of the bacterial microorganisms within the biofilm, although PVP-I and H_2_O_2_ were both far superior to CHX in reducing the viable fungal load, likely arising from a level of tolerance exhibited by the fungal organism. Indeed, this is in line with what our research group has shown previously whereby 48-h mature biofilms of *C. albicans* are more tolerant to 0.05% CHX than PVP-I or H_2_O_2_ when grown in the hydrogel model system^52^.

A 3D co-culture tissue model was utilised for assessing the host response to the polymicrobial biofilms. This reconstructed, commercially available tissue model comprised of normal human keratinocytes cultured on an inert polycarbonate filter at the air-liquid interface, forming a multi-layered structure containing a peripheral layer of keratin, to mimic in vivo human epidermis. Although limitations exist in our model, in particular an absence of resident and infiltrating immune cells, underlying connective tissue, and damaged epidermal/dermal layers associated with chronic wounds, evidence of such 3D co-culture model systems are limited. Of the in vitro biofilm co-culture model systems that exist, studies primarily focus on assessing the response of host cells to planktonic or sessile microorganisms associated with normal skin and/or infections, including several treatment modalities. For example, a number of studies have looked at the response in human skin keratinocytes to microorganisms such as *S. aureus* and/or *P. aeruginosa*^53–55^, although these are restricted to monolayers of epithelial cells exposed to planktonic cells or biofilms. Conversely, 3D-like co-culture systems have been reported in the literature, similar to that described here, although most models are commonly comprised of a mixture of collagen substrata, fibroblasts, and keratinocytes grown at the air-liquid interface like those described by Carlson et al. (2008). Such models provide a substantial improvement over 2D culture systems, displaying a spatially organised 3D tissue with structural features similar to in vivo^56^ and have been used to study the inflammatory response to *S. epidermis*, *S. aureus*, or *Actinobacter baumannii*^26–30^. Of note, Redderson et al. (2019) developed a 3D skin model consisting of collagen, epidermal fibroblasts, and keratinocytes to assess the efficacy of antiseptics in reducing the bacterial burden of *S. aureus* and subsequent host response to the organism. The authors found that treatment of 1 ×10^9^ cells/mL *S. aureus*-infected tissue with 0.2% CHX and 1.0% PVP-I significantly reduced the bacterial burden by 7 and 4 log units, respectively. Both treatments also altered the host response at both the transcriptional and proteomic level. Interestingly, CHX decreased the expression of *IL6* and *IL8* when compared to the untreated controls, but at the protein level, IL-6 and IL-8 levels were still high, which likely coincided with the cytotoxic effects of the compound against the host tissue^29^. This may explain the Olink data presented here, by which the CHX-treated and untreated biofilms gave rise to similar inflammatory proteomic profiles in the RHE tissue. However, this is mere postulation as without appropriate antiseptic-only controls minus biofilm, we cannot conclude this. Future studies merit testing the cytotoxic and/or inflammatory effects of the antiseptics on this 3D RHE tissue model. Indeed this is of relevance as it has been shown elsewhere that CHX is widely considered to be cytotoxic to human skin cells^57–59^. Alternatively, there is evidence in the literature the CHX treatment can lead to retention of microbial DNA due to the biochemical properties of the compound^60^. Thus, regardless of treatment efficacy, retention of biofilm DNA could elicit an elevated inflammatory response in the tissue, one that is comparable to untreated biofilms. Nevertheless, this study highlights that clinicians must carefully consider the antiseptics in wound therapy as although some treatments may be effective in reducing the bioburden, some modalities could lead to cytotoxicity, elevated inflammation, and inadequate wound healing within the host tissue.

We have demonstrated here that 3D co-culture models can be used effectively to assess the efficacy of antiseptics against interkingdom biofilms and the subsequent inflammatory profile of skin epidermis following biofilm stimulation. Unique to this study, Olink proteomic technology was used to investigate how the RHE tissue responded to the untreated and treated biofilms. Olink has widely been used to study the host response in different inflammatory diseases as well as cancer, mostly in vivo, with over 500 publications reported for human studies at the time of writing. However, the use of Olink proteomics for in vitro studies are limited. Of note, one study assessed the inflammatory response of human umbilical vein endothelial cells (HUVECs) following exposure to heat-killed *Streptococcus pneumoniae* and *C. albicans*. The authors utilised proteomics to investigate interactions between *S. pneumoniae* or *C. albicans*-stimulated peripheral blood mononuclear cells and HUVECs^61^. To the authors knowledge, other host-pathogen studies using Olink technology do not exist. Although only 31 of the 92 “inflammation” panel of proteins were detected, a result that some of the proteins are cell-associated (membrane attached or intracellularly located) and not secreted, these results provide substantial scope for the use of such technology in deciphering the role of biofilm-host interactions at the protein level.

To conclude, we have developed a distinctive 3D co-culture system for studying the host response in skin epidermis to polymicrobial wound biofilms. Preliminary observations showed that “early” 24-hour biofilms were more pro-inflammatory to differentiated monocytes than complex “mature” 72-hour biofilms. We next demonstrated that the treatment modality of such biofilms can affect how the host responds, particularly at the protein level, regardless of anti-septic efficacy. Specifically, CHX-treated and untreated “early” biofilms gave rise to unique proteomic profiles in the RHE tissue following stimulation, whilst PVP-I and H_2_O_2_-treated biofilms were arguably more immuno-modulatory and may be more effective choices for clinicians as anti-biofilm wound washes. It would be of interest for future studies to investigate the response in the RHE tissue to more complex “mature” biofilms, especially given that persistent biofilms containing certain consortia of microorganisms can cause chronic wounds and prevent tissue healing in some patients. Even more so if the mature biofilms are more resilient to the treatments, due to the thicker ECM. Nevertheless, this study highlights a unique proof of concept model system that could be utilised for a range of wound-related biofilm studies and/or antimicrobial testing. This model system paves the way for preclinical studies aimed to improve the management of chronic wound infection.

## Methods

### Polymicrobial biofilm development

Previous microbiome analyses were used to guide the development of the complex polymicrobial chronic wound biofilm model^62^. The organisms selected for incorporation into the model contained a range of skin commensals, including aerobes and anaerobes, and a fungal element. Of all those organisms included, all genus and/or species have been identified in the microbiome of the wound. Such a consortia were selected using a comprehensive literature search of previous microbiome studies showing the diverse nature of chronic wounds, in particular in diseases such as diabetic foot ulcers^14,35,36,63–67^.

### Microbial growth and standardization

All microorganisms used in this study are shown in Table 1. Firstly, all isolates stored in Microbank® vials at -80°C were revived on solid agar for between 24 and 72 hours then 2-3 colonies are propagated into liquid media as stated in Table 1. Following the growth of all organisms, overnight cultures were washed twice by centrifugation (3,000 x g) and resuspended in 10 mL phosphate-buffered saline (PBS). All cultures were standardized and adjusted to 1 × 10^8^ cells/mL, using optical density at 550 nm or 600 nm for bacterial strains and counted using a haemocytometer for *C. albicans*. The OD value and wavelength used for each bacterial species were as follows; 0.6 at OD_600nm_ for *P. aeruginosa*, *S. aureus*, *S. hominis*, and *C. simulans*, 0.5 at OD_550nm_ for S. agalactiae, and 0.25 at OD_550nm_ for the five anaerobic organisms. All anaerobes were grown at 10% CO_2_, 10% H_2_, 80% N_2_, with relevant solid and liquid media pre-reduced for at least 2 hours prior to growth.

**Table 1:** The microbial strains used in the study.

Strain	Liquid media	Solid media	Incubation conditions	Primer sequences for molecular detection
*Candida albicans* SC5314	Yeast peptone dextrose broth	Sabouraud dextrose agar	Aerobic conditions, 30°C, overnight	F- GAGCGTCGTTTCTCCCTCAAACCGCTGG R- GGTGGACGTTACCGCCGCAAGCAATGTT
*Pseudomonas aeruginosa* PA14	Luria broth	Luria agar	Aerobic conditions, 37°C, overnight	F- GGGCGAAGAAGGAAATGGTC R- CAGGTGGCGTAGGTGGAGAA
*Staphylococcus aureus* Newman’s strain				F- ATTTGGTCCCAGTGGTGTGGGTAT R- GCTGTGACAATTGCCGTTTGTCGT
*Staphylococcus hominis* DSMZ 15614	Tryptic soy broth	Columbia blood agar with 5% horse blood	5% CO_2_, 37°C, overnight	F- TAGATGGATCTGAAACAGTAGTAT R- CCTTCAACAATACCAAATTCGTC
*Corynebacterium simulans* DSMZ 44415				F- GGGCTTGACATATAGCGGAT R- CGGGACTTAACCCAACATCT
*Streptococcus agalactiae* DSMZ 2134				F- GATACATAGCCGACCTGAG R- CCATTGCCGAAGATTCC
*Finegoldia magna* DSMZ 20470	Schaedler Broth	Fastidious anaerobe agar with 5% horse blood	Anaerobic conditions (10% CO_2_, 10% H_2_, 80% N_2_), 37°C, 72 h	F- ATGACAGTGGGATAGCCTCG R- CGAGCCCATCTATGACCGAT
*Prevotella buccalis* DSMZ 20616				F- GGGATGCGTCTGATTAGCTTGTT R- CTGCACGCTACTTGGCTGGTTC
*Porphyromonas asaccharolytica* DSMZ 20707				F- AGGAACCTTACCCGGGATTG R- AGCACCTACATAGAAGCCCC
*Anaerococcus vaginalis* DSMZ 7457				F- GGCTTGAGAGATGAAAGGGA R- CTTTCGTACCTCAGAGTCAG
*Peptoniphilus gorbachii* DSMZ 21461				F- TGGTTTAATTCGAAGCAACG R- ATCTCACGACACGAGCTGAC

The culture media and conditions for the bacterial and fungal species are detailed here. For *C. albicans* growth in liquid media, suspensions were gently rotated at 150 rpm. The forward and reverse primer sequences are also shown for compositional analysis by qPCR. All primer sequences were designed in-house and tested for primer specificity.

### Hydrogel model system preparation and biofilm formation

**Fig. 7:**
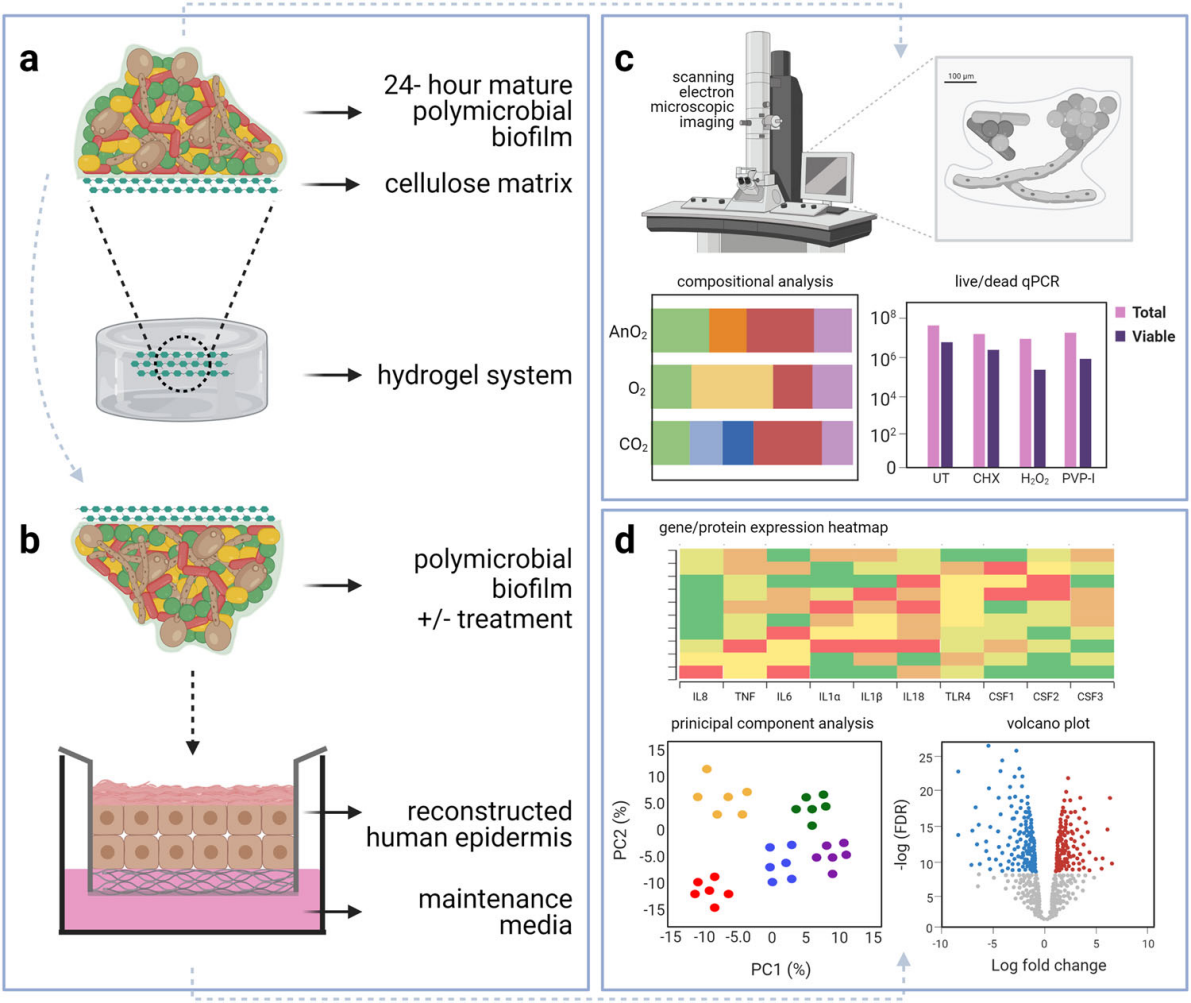
The two co-culture systems utilized in this study. 11-species biofilm was formed on and throughout the porous cellulose matrix substrate using the serum-containing hydrogel system. These biofilms were also treated with CHX, H_2_O_2_ or PVP-I following growth on the hydrogel (**a**). For the co-culture set-up, the matured biofilms were removed from the hydrogel and transferred to the reconstructed human epidermis (**b**). Experimental outputs for the study are shown in panels **c**, **d**, for the biofilm and RHE tissue experiments, respectively. In brief, the main outputs for the biofilm part of the study involved scanning electron microscopic imaging, compositional analysis and live/dead qPCR. For the tissue part of the study, this included gene expression and proteomic analyses including presentation of data as heatmaps, volcano plots and/or principal component analyses.

To develop a system that supported the growth of complex biofilms, a hydrogel-supported cellulose matrix model was used as previously described by our research group^18^. The substrate used for the study was cellulose matrix (CM), to mimic wound dressing. To form the biofilm, all 11 microbial species were added simultaneously to one universal containing sections of CM (1.25 cm² in diameter) then cultured on the hydrogel system as shown in Fig. 7a. The standardized microbial cultures were diluted from 1 ×10^8^ CFU/mL to a final concentration of either 1 ×10^6^ CFU/mL (for preliminary co-culture with THP-1 cells) or 1 ×10^7^ CFU/mL (for treatment and skin epidermis co-culture studies) in sterile PBS. For all experiments, a total of 1 mL of either 1 ×10^6^ CFU/mL or 1 ×10^7^ CFU/mL for each individual microorganism was added for each biofilm inoculum.

The suspension was initially incubated aerobically, in 5% CO_2_ or anaerobically at 37 ^°^C with gentle agitation for 2 h, to allow attachment of the microbes to the matrix. The CM was then placed on top of the hydrogel surface and incubated in the above conditions at 37 ^°^C for up to 9 days, ensuring the whole matrix was in contact with the agarose surface. The hydrogel was remoisturized with 200 µL of sterile water added periodically every second day. Appropriate control CM were added to the hydrogels minus microbial inoculation. Following biofilm formation, the CM was gently washed twice by immersion in sterile PBS to remove non-adherent cells.

### Biofilm treatment

For treatment of the biofilms, three conventional anti-biofilm wound therapeutics were selected. These treatments, hydrogen peroxide (H_2_O_2_), povidone iodine (PVP-I) and chlorhexidine (CHX) (all Merck Life Sciences, Gillingham, UK), were prepared fresh in sterile ddH_2_O prior to use. The following clinically relevant concentrations of each compound were used; 3% H_2_O_2_, 10% w/v PVP-I, and 0.05% CHX. A total of 1 mL of each therapeutic was be added to the biofilms, and hydrogels incubated for a further 24 hours under anaerobic conditions. For all treatment studies, appropriate untreated biofilms were used as controls (these biofilms received sterile water only, minus therapeutic). After treatment, the CM was washed twice by immersion in sterile PBS to remove excess therapeutic.

### Compositional analysis and live/dead qPCR

Experimental outputs for the biofilm studies are schematically depicted in Fig. 7c. Following biofilm formation and treatment, CM was removed carefully and transferred to a bijoux containing 1 mL of sterile PBS using tweezers then sonicated at 35 kHz for 10 minutes to remove the biomass. The live/dead quantification method was used to allow discrimination of viable and total bacteria and fungi in the samples, with the method followed as previously described^68^. For compositional analysis, the above propidium monoazide treatment steps were omitted and the following sonication, samples were used directly for DNA extraction.

In brief for the live/dead qPCR methodology, 5 μl of 10 mM propidium monoazide (a compound that will only penetrate cells with compromised cell membranes) was added to 500 µL of the above sonicate. The other 500 μl of sample would act as negative control minus propidium monoazide addition, giving the total colony-forming equivalents (CFE) per biofilm. All samples were then incubated in the dark at room temperature for 10 minutes to allow for uptake of propidium monoazide into the dead cells. Samples were then placed on ice and exposed to a 650 W halogen light positioned approx. 20 cm away for 5 minutes. Following exposure, microbial DNA was extracted from biofilms. For compositional analysis, the above propidium monoazide treatment steps were omitted and following sonication, samples were used directly for DNA extraction.

### DNA extraction and qPCR

Microbial DNA was extracted using the QIAamp DNA Mini Kit, according to manufacturer’s instructions, with small amendments. Samples were also mechanically disrupted using 0.5 mm glass beads and homogenised for 90 s using BeadBug™ microtube homogeniser. To produce bacterial and fungal standard curves, single species pure cultures were standardised to 1 ×10^8^ CFU/mL in sterile PBS, serially diluted to 1 ×10^3^ CFU/mL and DNA extracted in the same way.

SYBR® green-based quantitative PCR was used to determine the total and live CFE/ml for 16 s (all bacteria) and 18 s (total *C. albicans*) in the biofilms following treatment, and either genus or species-specific primers for compositional analysis. The list of primer sequences used for compositional analysis is shown in Table 1. All genus or specific primer sequences were designed in-house, with melting curve analysis performed to ensure a single peak, which was indicative of primer specificity. For 16 s and 18 s, the same primer sequences were used as described elsewhere^68^. These were as follows; 16 S, 5’-CGCTAGTAATCGTGGATCAGAATG-3’ for forward primers and 5’-TGTGACGGGCGGTGTGTA-3’ for reverse primers. For 18 S, 5’-CTCGTAGTTGAACCTTGGGC-3’ for forward primers and 5’- GGCCTGCTTTGAACACTCTA-3’ for reverse primers. The thermal profile for genus or species-specific primer sets were as follows; a 2 min initial denaturation step at 95 °C, followed by an amplification cycle of 40 cycles of 95 °C for 25 secs, the appropriate annealing temperature (60.0 °C) for 35 s, and a 65 s extension at 72 °C. For the 16 s and 18 s primers, the PCR reaction was 95 °C for 2 minutes, 40 cycles of 95 °C for 3 s followed by 55 °C for 30 s. All samples were run in duplicate and a negative control well containing Fast SYBR® Green Master Mix and each primer set minus microbial DNA was included on every plate to rule out the presence of contamination. Data presented as mean composition; averaged values from *n* = 3 from two separate experiments (e.g., a total of 6 replicates).

### Scanning electron microscopy

To assess the of the organization of the CM and ultrastructure of the biofilm formed on it, scanning electron microscopy (SEM) was used. For this, the samples were prepared as previously described^69^ and imaged using a JEOL JSM-6400 SEM machine (JEOL Ltd, Hertfordshire, UK) at magnification of ×2500.

### THP-1 co-culture

Frozen stocks of THP-1 cells (1 ×10^6^ cells/mL; Invitrogen, Paisley, UK), a leukemic pro-monocyte cell line was revived from storage in liquid nitrogen prior to experimental use. Cryovials were thawed at 37 °C before transferring into a 70 cm^2^ cell culture flask and grown as previously described^70^. For differentiation of THP-1 cells, 100 nM of 1α,25-dihydroxyvitamin D3 (vitamin D3, Enzo Life Sciences, Farmingdale, NY, USA) was prepared in RPMI-1640 media containing 10% FBS and cells incubated for 48 hours at 37°C in 5% CO_2_ to enable differentiation. Once differentiated, supernatants containing non-adhered cells were removed, adherent cells washed with PBS then utilized as below.

For the conditioning of cell culture media, the CM containing wound biofilms of 24-h, 48-h, and 72-h maturity grown as above, were removed from the hydrogel system, and placed into 2 mL of RPMI-1640 media containing 10% FBS. These were then allowed to incubate for 24 h at 37 °C under anaerobic conditions. Following incubation, the media was removed and filter sterilised using a 0.22 μm filter to remove any dispersed bacterial or fungal cells. The conditioned media was diluted 1:4 with FBS-supplemented RPMI-1640 media to a final volume of 1 mL, and added to differentiated THP-1 cells, then incubated for 24 h at 37 °C in 5% CO_2_.

### 3D co-culture skin epidermis model

Reconstituted human epidermis (RHE) used for 3D co-culture experiments was purchased from Episkin (Skin Ethic; Episkin, Lyon, France). RHE was formed from healthy human keratinocytes cultured on an inert polycarbonate filter at the air-liquid interface, in a chemically defined medium grown to 17-day maturity. Upon arrival and prior to experimental set-up, RHE was incubated with 1 mL of maintenance medium in 24-well plates for 24 h with 5% CO_2_ at 37 °C. The maintenance medium was replaced (1 mL), and then the co-culture system was set up. A similar co-culture set-up was utilized as described previously by the group^71,72^ and as highlighted in Fig. 7b. Small amendments were made to the model, to allow the incorporation of biofilms grown on CM. In brief, following initial 24 h incubation in maintenance medium, biofilms formed on CM were gently layered on top of the RHE tissue prior to the addition of 100 µL sterile PBS to maintain moisture of the CM substrate and biofilm. Biofilms were formed and treated on the hydrogel system as described above, then washed twice in sterile PBS and carefully removed using sterilized tweezers to ensure biofilm was not disrupted. For negative controls, sterile CM minus biofilms was used with 100 µL sterile PBS. For positive controls, phorbol 12-myristate 13-acetate (PMA) was used to stimulate the tissue. For this, 50 ng/ml of the compound was prepared in 100 µL sterile PBS and added directly to the RHE tissue. All co-cultures were then incubated for a further 24 h at 5% CO_2_, 37 °C. Key experimental outputs for the RHE study are depicted in Fig. 7d.

### Histological assessment of RHE tissue

Following co-culture, RHE tissue was carefully cut from the 0.5 cm^2^ insert using a 19-gauge needle then fixed in 10% neutral buffered formalin prior to embedding in paraffin. A Finesse ME + microtome was used to cut 2-μm sections, and these were stained with hematoxylin and eosin to assess the histology of the tissue.

### Gene expression profiling of THP-1 cells and RHE tissue

For THP-1 cells and RHE tissue, these were processed as previously described for RNA extraction^71^. All RNA was extracted using the RNeasy minikit according to the manufacturer’s instructions (Qiagen Ltd., Manchester, UK) and quantified using a NanoDrop 1000 spectrophotometer. RNA was first standardized to 100 ng/μL then converted to complementary DNA (cDNA) using the high-capacity RNA to cDNA kit per the manufacturer’s instructions. A custom-designed RT_2_ Profiler PCR Array (Qiagen Ltd., Manchester, UK) was used to assess the gene expression of a panel of pro- and anti-inflammatory cytokine/chemokine genes following stimulation of either the differentiated THP cells or RHE tissue. For the THP-1 cell co-culture the following genes of interest were used; *IL-1α*, *IL-1β*, *IL-6*, *TNFα*, *CSF2*, *CSF3*, *IL-8*, *CXCL1*, *CXCL3*, *CXCL5*, *CCL1*, and *GAPDH*, with the latter serving as the house-keeping gene. Compilation of the panel for the RHE tissues was guided by proteomic results along with the inclusion of “classic” markers of inflammation. The following 23 genes were selected for the customized array in addition to a housekeeping gene, *GAPDH*; *IL33*, *CXCL8*, *CCL7*, *CFS2*, *CASP8*, *TNF, CFS3*, *TLR4*, *IL18*, *CD40*, *KITLG*, *AXIN1*, *CXCL5*, *ADA*, *IL1B*, *EIF4EBP1*, *IL20RA*, *VEGFA*, *IL1A*, *SIRT*, *STAMBP*, *CFS1*, and *IL6*. For both qPCRs, the following thermal cycles were used on the MxProP quantitative PCR machine; 10 min at 95 °C, followed by 40 cycles, where 1 cycle consisted of 15 s at 95 °C and 60 s at 60 °C. Data were assembled using MxProP 3000 software (Stratagene, Agilent, Stockport, UK).

### Proteomic analyses

#### Olink technology

For all co-culture models, spent tissue supernatant was saved for Olink technology, which utilises a proximity extension assay (PEA) methodology, combining antibody-based immunoassays with a polymerase chain reaction (PCR), alongside qPCR or next-generation sequencing for protein detection^73^. For this, 50 µL of each supernatant was directly added to a 96-well PCR plate and sealed using an adhesive PCR plate cover. The plates were then stored at -80^o^C prior to shipment to Olink Proteomics AB (Uppsula, Sweden). The “inflammation” panel of biomarkers was selected to assess the protein response in the skin epithelium following biofilm stimulation. For this, a total of 92 biomarkers were assessed, with samples randomized across the plate. A list of all 92 biomarkers can be found at the following link (https://www.olink.com/products-services/target/inflammation/). Expression of all markers were provided by Olink Proteomics AB as NPX units, with varying threshold levels for each marker and these can be found as a supplementary data attached with this manuscript (Supplementary Fig 9). In total, 35 markers were detectable in the samples provided, with the remaining 57 markers below the limit of detection (LOD). Of these, 31 markers were expressed at significantly different levels amongst the groups. The LOD was determined as 3 standard deviations above background. It is noteworthy that media-only controls (minus tissue) supplied by us were below the LOD for all 92 markers. Method validation for the “inflammation” panel including performance characteristics can be viewed using the article number 95302 at https://www.olink.com/resources-support/document-download-center/. Analyses of the proteomic data was completed using Olink insights stat analysis online tool (https://www.olink.com/products/insights-stat-analysis/). In brief, for the hierarchical clustering performed on the 31 significant and detectable markers, all distributions in marker expression were centered at 0 and scaled to have a standard deviation of 1. Hierarchical clustering was then based on centered and scaled NPX values for both samples and proteins to determine row and column ordering.

#### ELISA methodology

To determine the level of IL-8 released by THP-1 cells; human IL-8 Standard TMB ELISA kits (Peprotech, London, UK) were used according to the manufacturer’s instructions. To corroborate some of the observations from the Olink proteomics data for the RHE tissue stimulation, three ELISAs were selected. These were VEGFA, IL-8, and IL-18 (Bio-Techne Ltd., Abingdon, UK). The methodology followed was according to that described in the manufacturer’s instructions. For all ELISAs, the expression of each protein was calculated according to a standard curve generated from known protein concentrations.

#### Statistical analyses

Graphs were compiled, and data analysed using GraphPad Prism (version 9; GraphPad Software Inc., La Jolla, USA) unless stated otherwise. For Olink analyses, data was analysed using the Olink insights stat analysis online tool (https://www.olink.com/products/insights-stat-analysis/) as discussed above. Principal component analysis graphs were compiled in Past4 (https://www.nhm.uio.no/english/research/infrastructure/past). For statistical analyses, normally distributed data as per Gaussian distribution was analysed by two-tailed Student’s t-test to compare the means of two samples or one-way analysis of variance (ANOVA) to compare the means of more than two samples, and Tukey’s post-test was applied to the *p* value to account for multiple comparisons of the data. Where appropriate, for non-normally distributed data, the Kruskal-Wallis test was used. All statistical analyses for qPCR data were completed on ΔCT values, although data is presented as Log_2_ fold change. For Olink data, to generate volcano plots assessing log fold change in protein concentration, all data was first corrected for multiple comparisons using the Benjamini–Hochberg false discovery rate (FDR) of 5%. Statistical significance was achieved if *p < 0.01.

### Reporting Summary

Further information on research design is available in the Nature Research Reporting Summary linked to this article.

## Supplementary information

Supplementary File

Reporting Summary

## Data Availability

The raw data that supports the Olink proteomic data in this study is available as supplementary data (Supplementary Fig 9). All other data that support the findings of this study are available from the corresponding author upon reasonable request.
