# Supplementary material for: Assessing the inflammatory response to in vitro polymicrobial wound biofilms in a skin epidermis model
Source: NPJ Biofilms Microbiomes. 2022 Apr 7;8:19. doi: 10.1038/s41522-022-00286-z (PMC8991182; doi:10.1038/s41522-022-00286-z)
Supplement: Supplementary file 1 — Supplementary File [file 41522_2022_286_MOESM1_ESM.docx]

***Assessing the inflammatory response to in vitro polymicrobial wound biofilms in a skin epidermis model***

**Jason L Brown, Eleanor Townsend, Robert D Short, Craig Williams, Chris Woodall, Christopher J Nile, Gordon Ramage**

**

**

**Supplementary Figure 1 – Scanning electron microscopic imaging of the uninoculated cellulose matrix (CM).** Control CM minus microbial inoculation was processed and imaged using scanning electron microscopy at a magnification of x 1000. Imaging highlights the fibrillar network of the CM substratum.

**
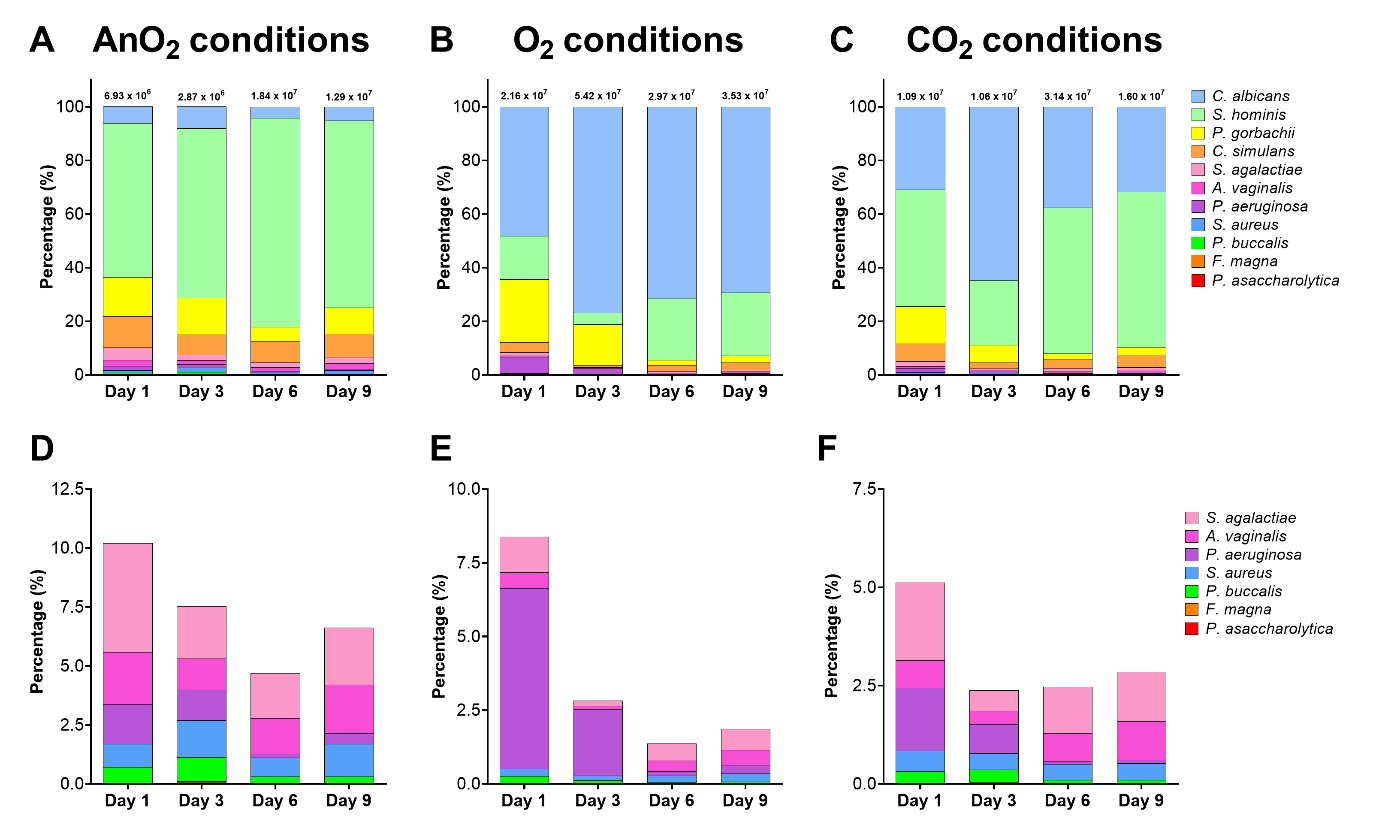
**

**Supplementary Figure 2** – **Compositional analysis of the 11-species biofilms grown under anaerobic (AnO_2_) aerobic (O_2_) and 5% CO_2_ conditions**. A total of 10 bacterial species and 1 fungal species were cultured under AnO_2_ (A), O_2_ (B) and 5% CO_2_ (C) conditions for a total of 9 days. Lower panels show the percentage composition of the microorganisms found in lower proportions in the three biofilm models (AnO_2_; D, O_2_; E and CO_2_; F). *F. magna* and *P. asaccharolytica* were found in very low proportions in all biofilm models (<0.01%) although these increased in % when grown under anaerobic conditions. On days 1, 3, 6 and 9, biofilms were sonicated, and DNA extracted. Compositional analysis was achieved using species or genus specific primer sets and qPCR. Data is presented as mean % composition from a total of 2 independent experiments, containing 3 replicates in each experiment.


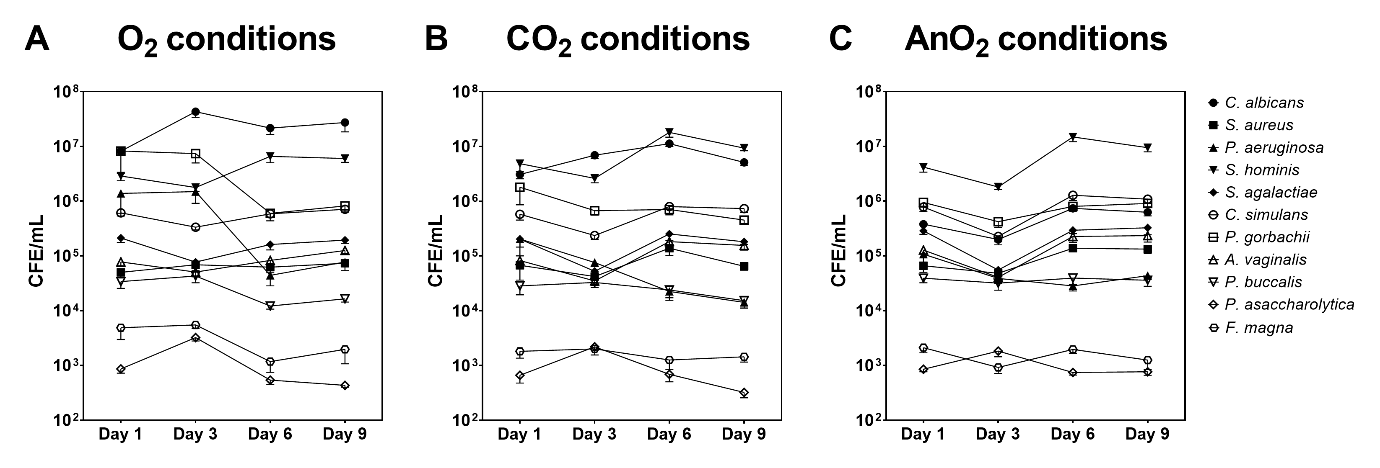


**Supplementary Figure 3 – Line graphs depicting the reproducibility of the biofilm models of differing maturity when grown in anaerobic (AnO_2_), aerobic (O_2_) and 5% CO_2_ conditions.** Polymicrobial biofilms were grown for a total of 9 days under three different environmental conditions (AnO_2_), aerobic (O_2_) and 5% CO_2_. On days 1, 3, 6 and 9, compositional analysis of the biofilms was compared. Data is presented as mean + SD from a total of 2 independent experiments, containing 3 replicates in each experiment.

**
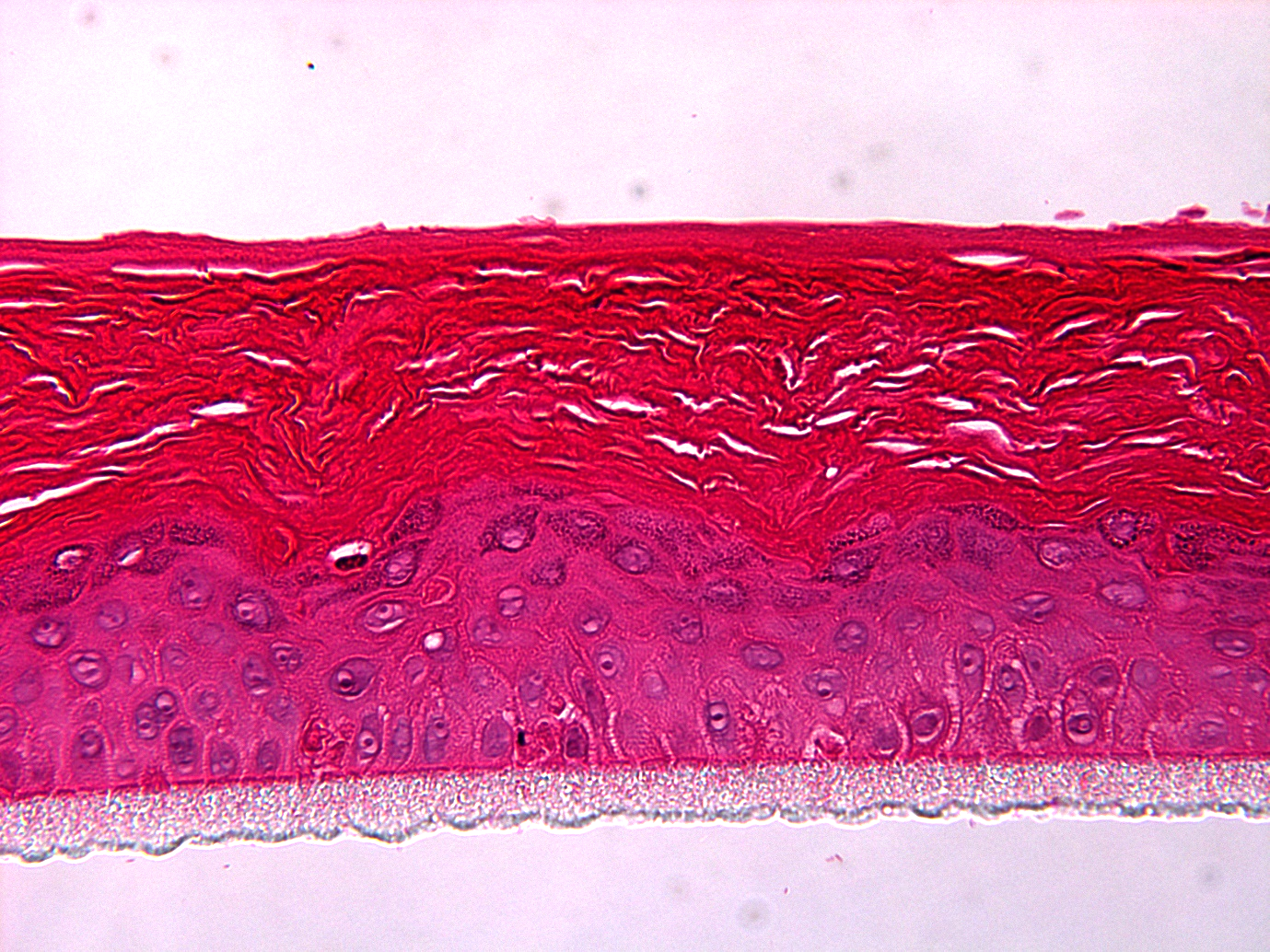
Supplementary Figure 4** – **Histological assessment of the 3D reconstructed human epidermis (RHE) used in this study**. Haematoxylin and eosin staining of an unstimulated RHE, highlighting the multi-layered morphology of the keratinised tissue mimicking skin epidermis *in vivo*. Image shown was taken at x25 magnification.


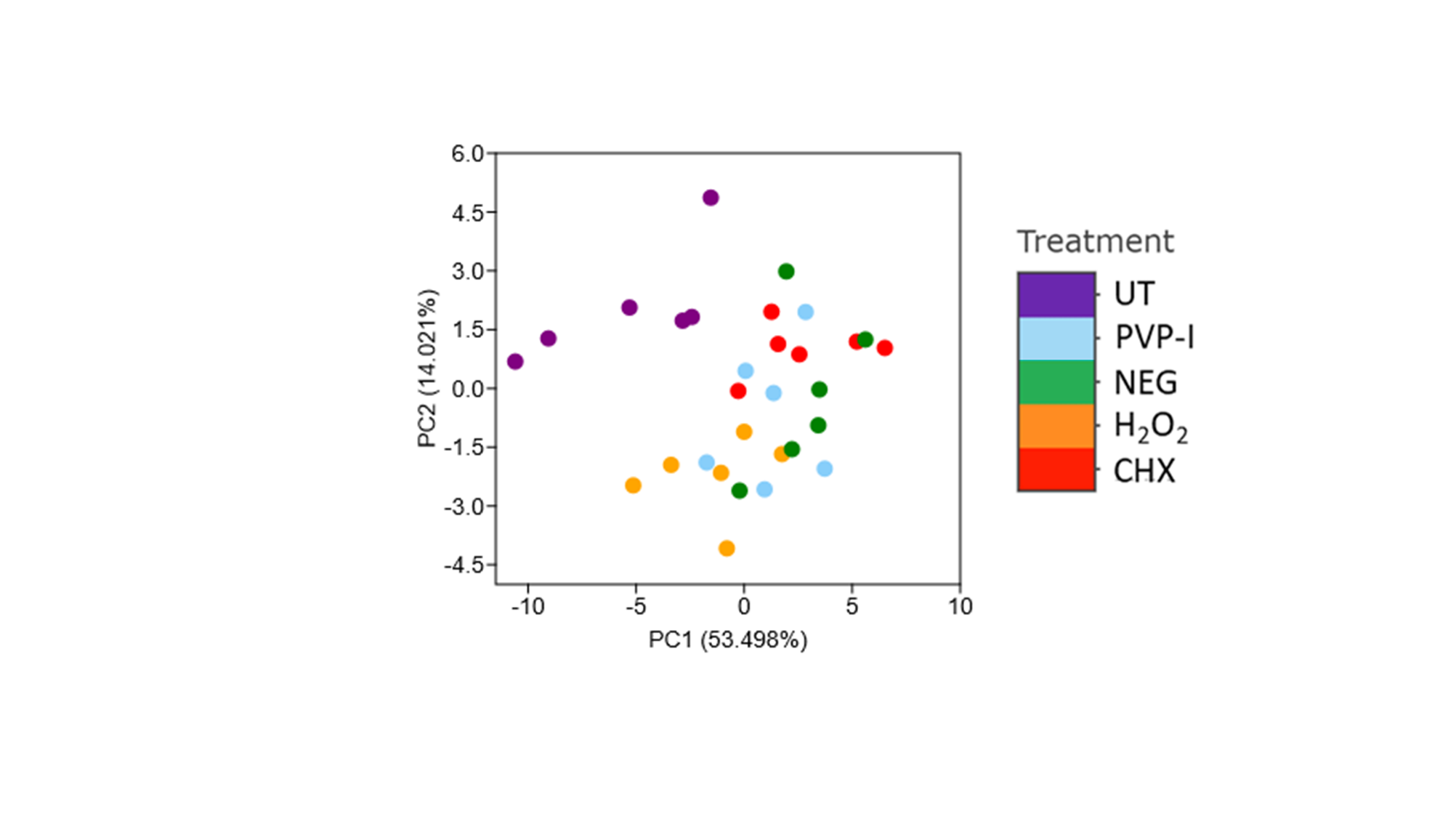


**Supplementary Figure 5 – Principal component analysis (PCA) plot of the transcriptional response in the RHE tissue following stimulation.** PCA plot depicting the clustering of RHE tissue samples following no stimulation, or stimulation with treated and untreated biofilms, based on their transcriptional response. Positive controls (PMA-stimulated tissue) were removed from analysis to highlight similar clustering of data sets. Graph was generated using Past4.

**
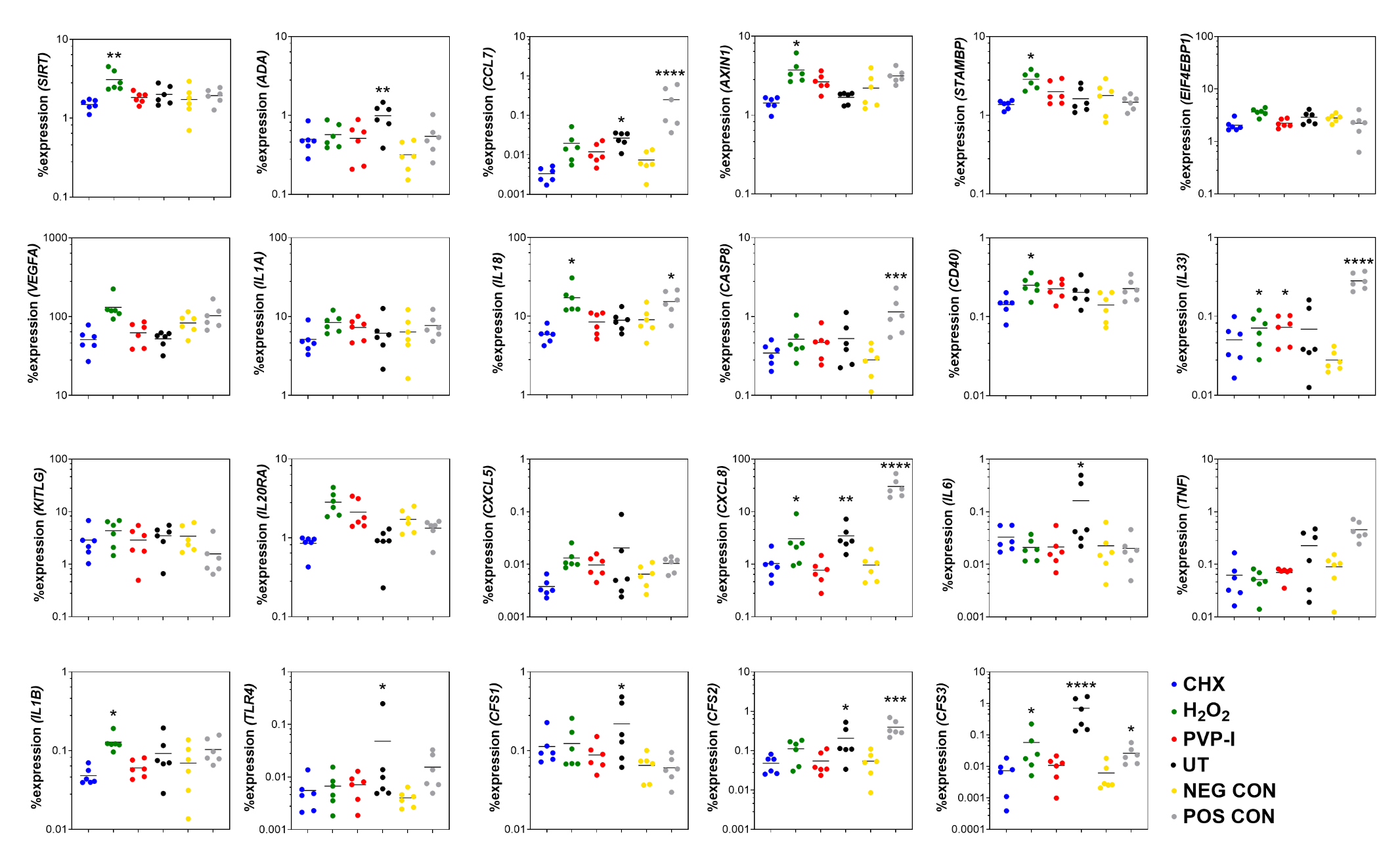
**

**Supplementary Figure 6 (continued)** - **Expression profiles of the RHE tissue following stimulation with untreated and treated biofilms**. A total of 23 genes were assessed using a custom RT2 qPCR array. The expression profile of the genes is shown as % expression relative to the house keeping gene, GAPDH. Data points are representative of individually stimulated tissues, a total of 6 from two independent experiments. Statistical significance values shown as *p<0.05, **p<0.01, ***p<0.001 and ****p<0.0001 and calculated all relative to the unstimulated tissue controls (NEG CON).

**
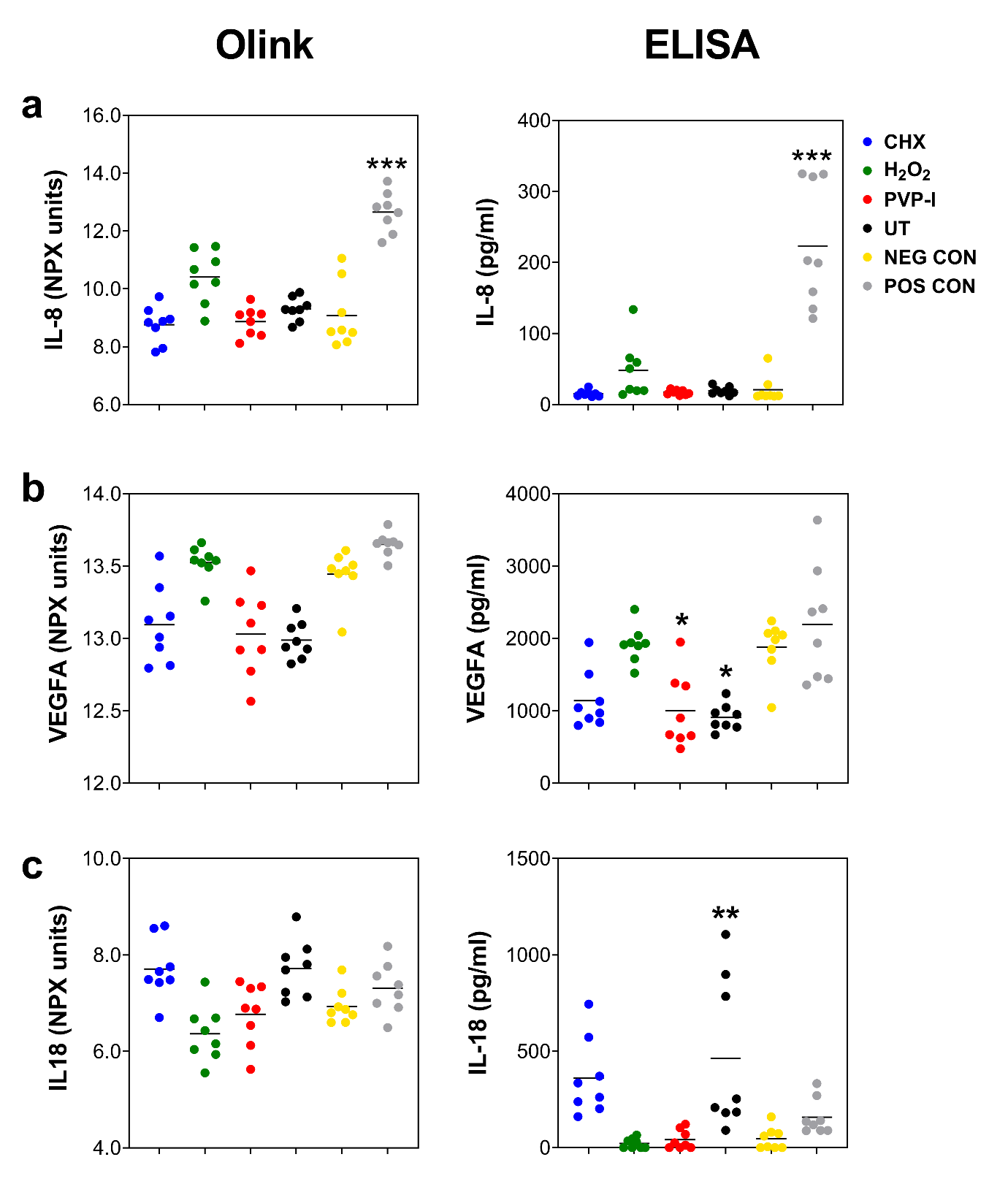
**

**Supplementary Figure 7** – Comparison of protein levels in spent culture media from the RHE tissue following stimulation with treated and untreated biofilms. Three proteins, IL-8. VEGFA and IL-18 were detected in the spent media from RHE tissue using two different methodologies, Olink proteomic technology and standard commercial ELISAs. Data points are representative of individually stimulated tissues, a total of 8 from two independent experiments. Statistical significance values shown as *p<0.05, **p<0.01 and ****p<0.0001 and calculated all relative to the unstimulated tissue controls (NEG CON).


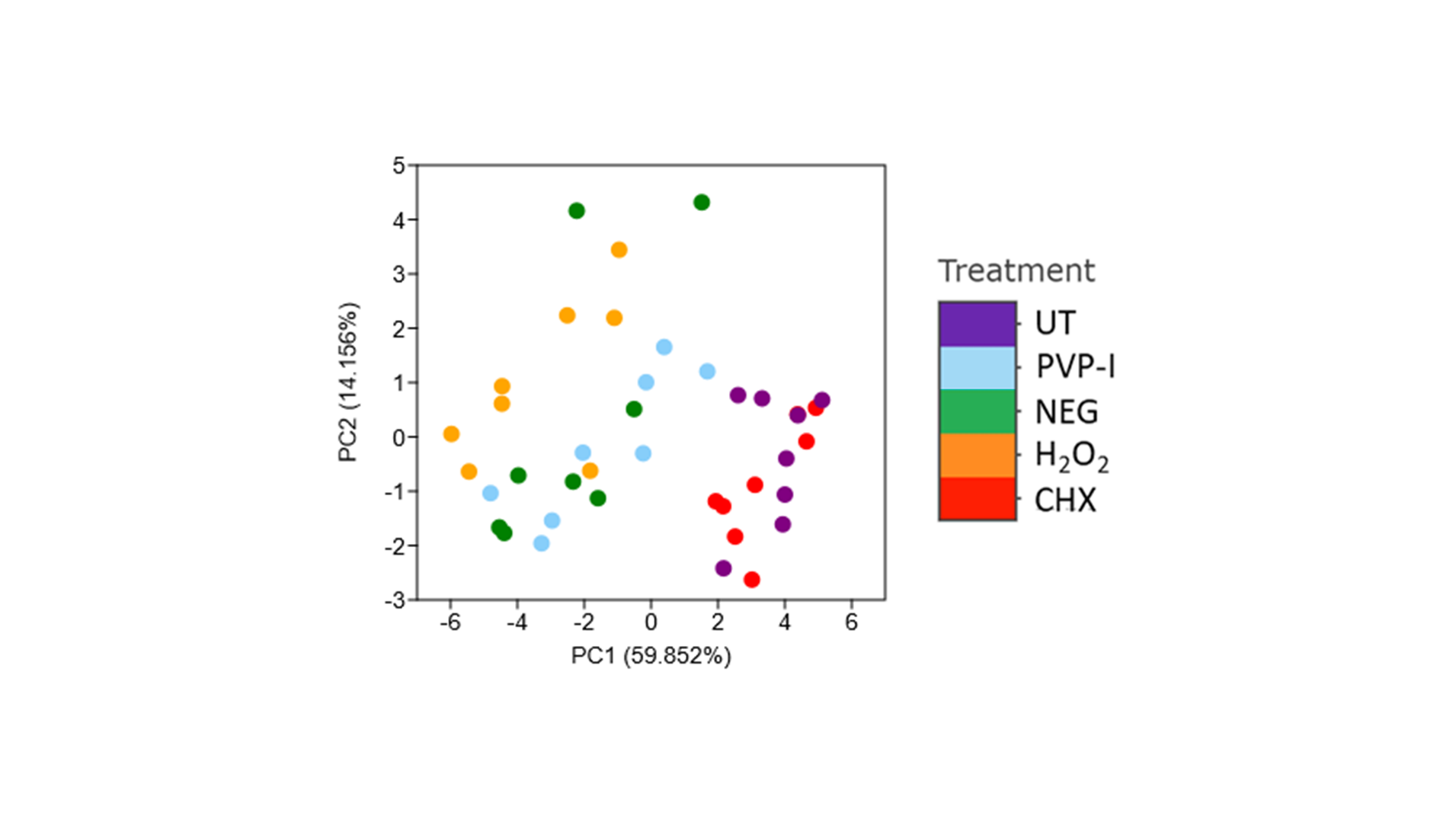


**Supplementary Figure 8 – Principal component analysis (PCA) plot of the proteomic response in the RHE tissue following stimulation.** PCA plot depicting the clustering of RHE tissue samples following no stimulation, or stimulation with treated and untreated biofilms, based on their proteomic response as assessed using Olink technology. Positive controls (PMA-stimulated tissue) were removed from analysis to highlight similar clustering of data sets. Graph was generated using Past4.

**
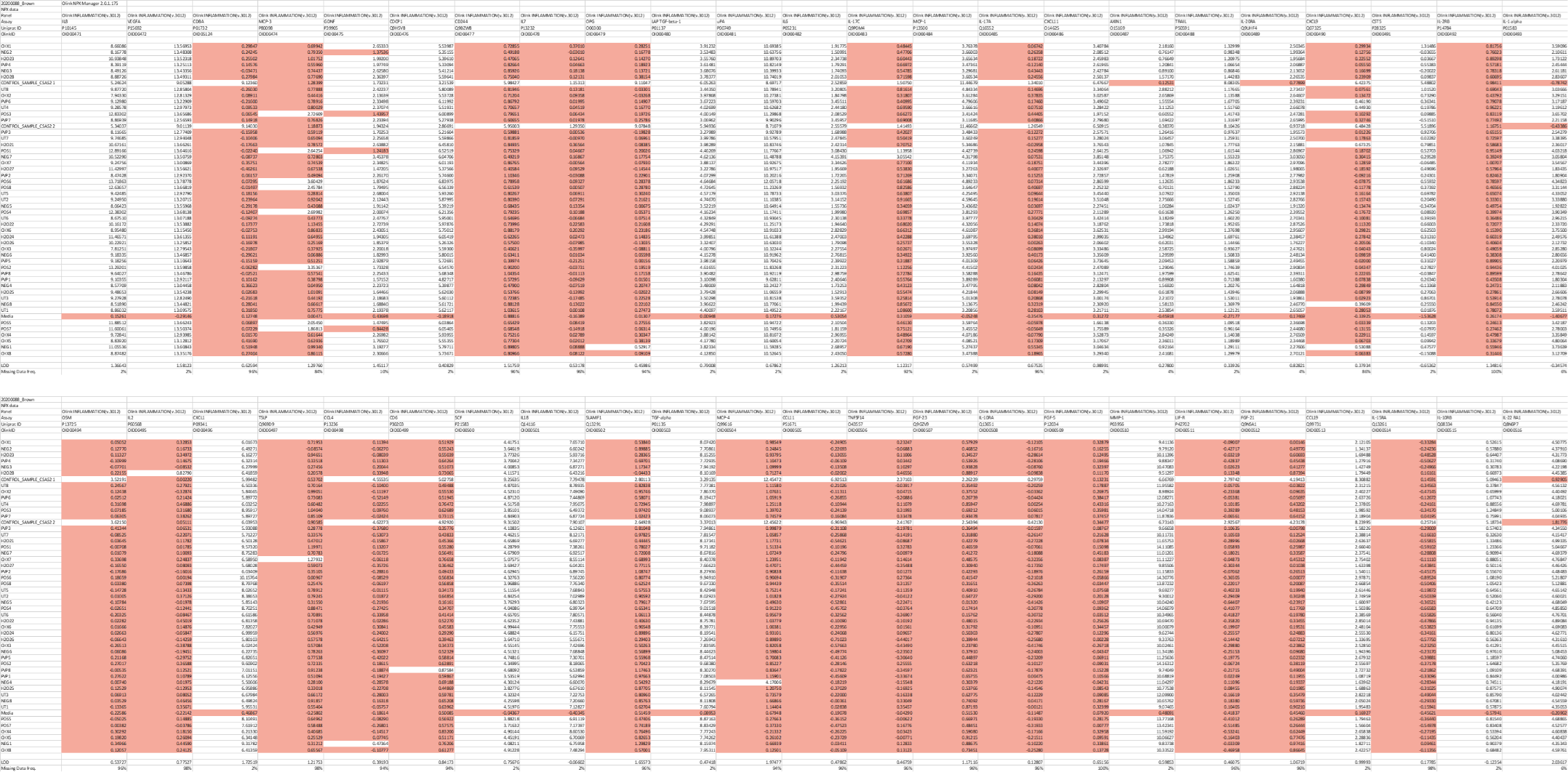
**


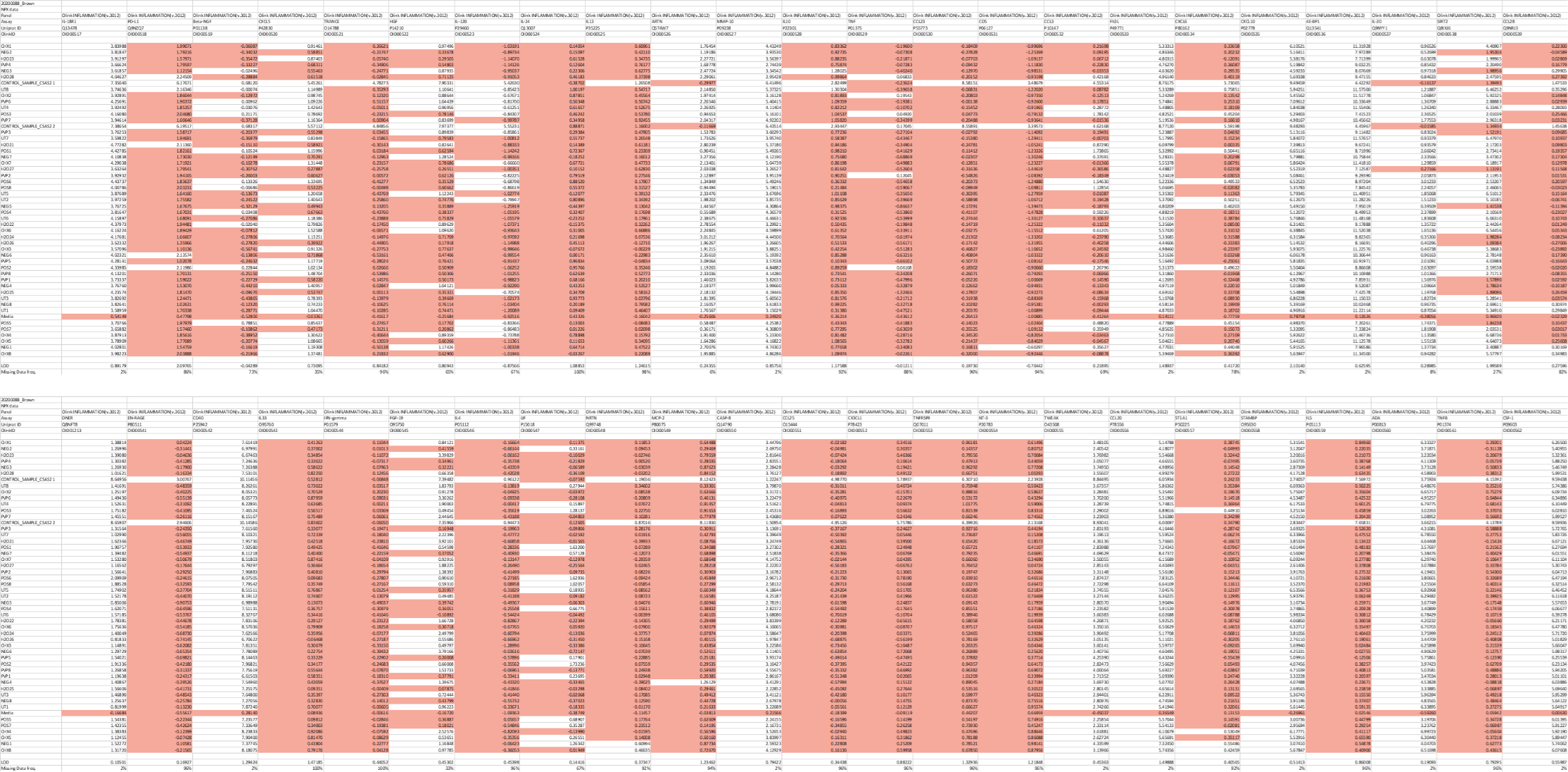


**Supplementary Figure 9 – Olink raw data.** All NPX values for the 92 proteins in the Olink “inflammation” panel. Detection of all proteins passed quality check assurance as assessed via the Olink proteomic technology. Red highlighted cells depict proteins that were below the limit of detection for the assay. Raw excel data is available from the authors upon reasonable request.
