# Supplementary material for: Assessing the inflammatory response to in vitro polymicrobial wound biofilms in a skin epidermis model
Source: NPJ Biofilms Microbiomes. 2022 Apr 7;8:19. doi: 10.1038/s41522-022-00286-z (PMC8991182; doi:10.1038/s41522-022-00286-z)
Supplement: Supplementary file 2 — Reporting Summary [file 41522_2022_286_MOESM2_ESM.pdf]

nature portfolio

Corresponding author(s): Professor Gordon Ramage

Last updated by author(s): Feb 6, 2022

Reporting Summary

Nature Portfolio wishes to improve the reproducibility of the work that we publish. This form provides structure for consistency and transparency in reporting. For further information on Nature Portfolio policies, see our Editorial Policies and the Editorial Policy Checklist.

Statistics

For all statistical analyses, confirm that the following items are present in the figure legend, table legend, main text, or Methods section.

n/a	Confirmed	
☐	☒	The exact sample size (n) for each experimental group/condition, given as a discrete number and unit of measurement
☐	☒	A statement on whether measurements were taken from distinct samples or whether the same sample was measured repeatedly
☐	☒	The statistical test(s) used AND whether they are one- or two-sided<br>*Only common tests should be described solely by name; describe more complex techniques in the Methods section.*
☒	☐	A description of all covariates tested
☐	☒	A description of any assumptions or corrections, such as tests of normality and adjustment for multiple comparisons
☐	☒	A full description of the statistical parameters including central tendency (e.g. means) or other basic estimates (e.g. regression coefficient) AND variation (e.g. standard deviation) or associated estimates of uncertainty (e.g. confidence intervals)
☒	☐	For null hypothesis testing, the test statistic (e.g. F, t, r) with confidence intervals, effect sizes, degrees of freedom and P value noted<br>*Give P values as exact values whenever suitable.*
☒	☐	For Bayesian analysis, information on the choice of priors and Markov chain Monte Carlo settings
☒	☐	For hierarchical and complex designs, identification of the appropriate level for tests and full reporting of outcomes
☒	☐	Estimates of effect sizes (e.g. Cohen's d, Pearson's r), indicating how they were calculated

Our web collection on statistics for biologists contains articles on many of the points above.

Software and code

Policy information about availability of computer code

Data collection	no software was used.
Data analysis	no software was used.

For manuscripts utilizing custom algorithms or software that are central to the research but not yet described in published literature, software must be made available to editors and reviewers. We strongly encourage code deposition in a community repository (e.g. GitHub). See the Nature Portfolio guidelines for submitting code & software for further information.

Data

Policy information about availability of data

All manuscripts must include a data availability statement. This statement should provide the following information, where applicable:

- Accession codes, unique identifiers, or web links for publicly available datasets
- A description of any restrictions on data availability
- For clinical datasets or third party data, please ensure that the statement adheres to our policy

The data that support the findings of this study are available from the corresponding author upon reasonable request.

Field-specific reporting

Please select the one below that is the best fit for your research. If you are not sure, read the appropriate sections before making your selection.

☒ Life sciences ☐ Behavioural & social sciences ☐ Ecological, evolutionary & environmental sciences

For a reference copy of the document with all sections, see nature.com/documents/nr-reporting-summary-flat.pdf

Life sciences study design

All studies must disclose on these points even when the disclosure is negative.

Sample size	As these were solely in vitro experiments no sample size calculation was required.<br>For all parts of the study, experiments were repeated at least once (e.g., biological repeats) with at least 3 technical replicates per variable in each group (e.g., at least 6 individual replicates from 2 separate experiments were gathered). As with similar studies, these number of replicates allow us to assess biological and technical variability, whilst still having enough samples to ensure statistical differences in tested parameters could be observed, if necessary.
Data exclusions	No data was excluded from the analysis.
Replication	All attempts at replication were successful.
Randomization	Randomization of samples in our study was not required. All biofilm or tissue models generated were comparable and reproducible between technical replicates so there was no requirement for randomization of samples.
Blinding	This was a solely in vitro study and all data included was quantitative, so blinding of data was not required. No parts of this study involved subjective or qualitative data, therefore blinding of investigators in regards to data analysis was not needed.

Reporting for specific materials, systems and methods

We require information from authors about some types of materials, experimental systems and methods used in many studies. Here, indicate whether each material, system or method listed is relevant to your study. If you are not sure if a list item applies to your research, read the appropriate section before selecting a response.

Materials & experimental systems

n/a	Involved in the study	
☒	☐	Antibodies
☐	☒	Eukaryotic cell lines
☒	☐	Palaeontology and archaeology
☒	☐	Animals and other organisms
☒	☐	Human research participants
☒	☐	Clinical data
☒	☐	Dual use research of concern

Methods

n/a	Involved in the study	
☒	☐	ChIP-seq
☒	☐	Flow cytometry
☒	☐	MRI-based neuroimaging

Eukaryotic cell lines

Policy information about cell lines

Cell line source(s)	Reconstructed human epidermis generated from normal human keratinocytes
Authentication	No authentication was generated for the cell lines used, as the models were available commercially from Episkin.
Mycoplasma contamination	All tissue models supplied by Episkin are tested for Mycoplasma prior to shipment.
Commonly misidentified lines (See ICLAC register)	n/a
